# Supplementary material for: Closing the gap between palaeontological and neontological speciation and extinction rate estimates
Source: Nat Commun. 2018 Dec 7;9:5237. doi: 10.1038/s41467-018-07622-y (PMC6286320; doi:10.1038/s41467-018-07622-y)
Supplement: Supplementary file 1 — Supplementary Information [file 41467_2018_7622_MOESM1_ESM.pdf]

Supplementary Information

Closing the gap between palaeontological and neontological speciation and extinction rate estimates

Daniele Silvestro, Rachel C. M. Warnock, Alexandra Gavryushkina, Tanja Stadler

Supplementary figures

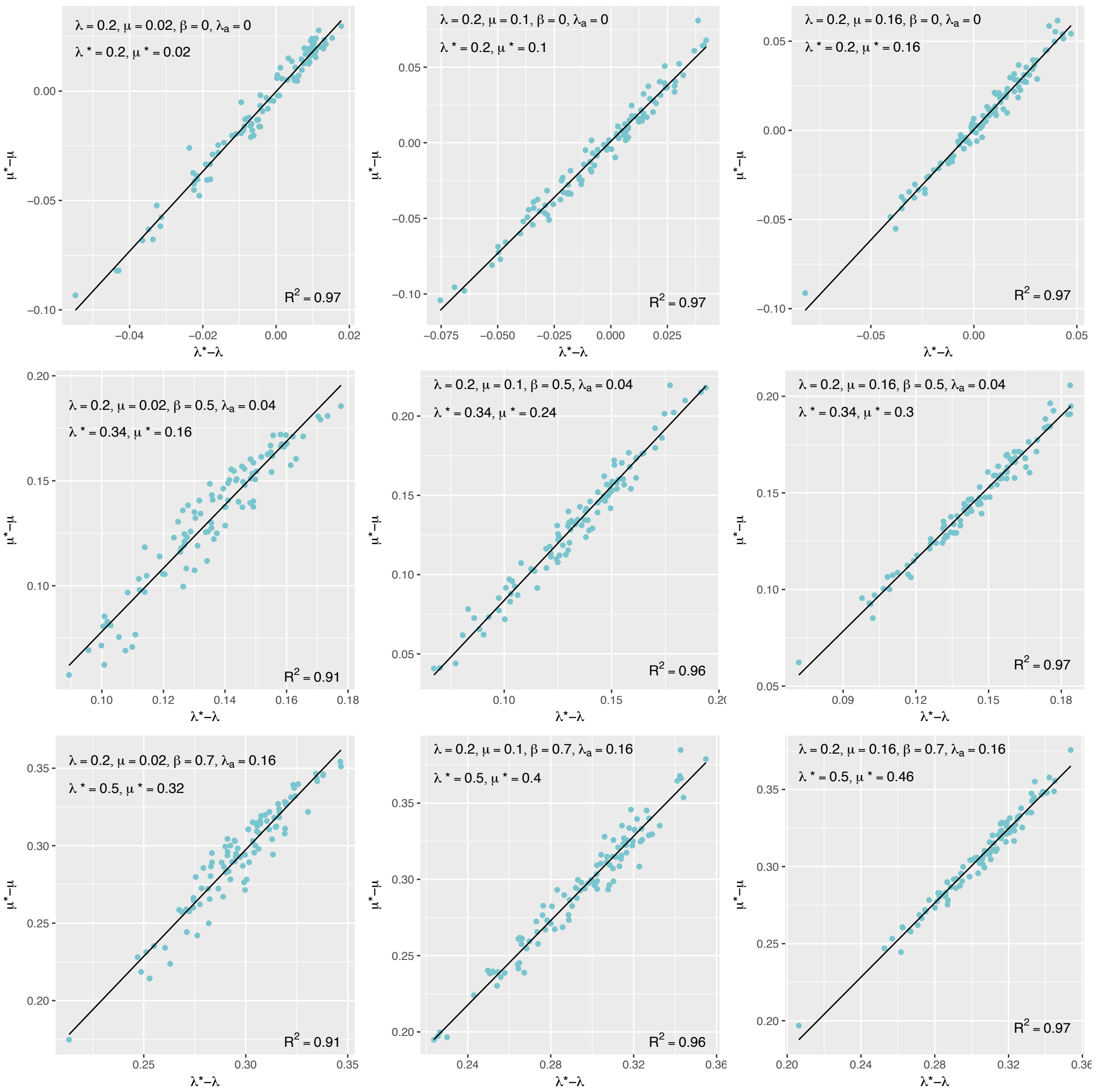

Supplementary Figure 1: The relationship between λ, μ, λ^* and μ^* according to Eqn. (5). Estimates are shown for simulations of fossil and phylogenetic data under the birth-death chronospecies model with different proportions of cladogenesis via budding or bifurcation, anagenetic speciation, and extinction without replacement.

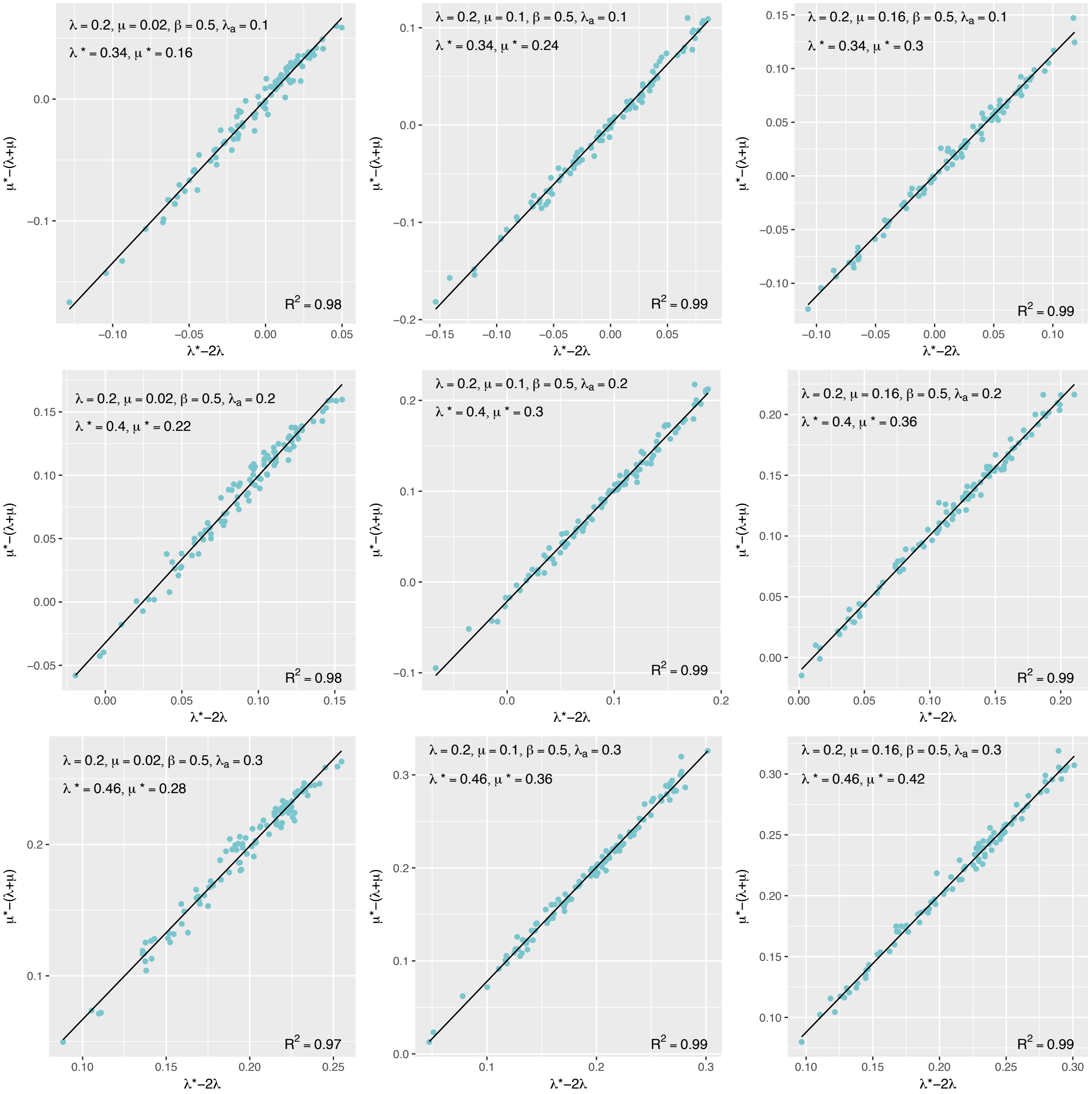

Supplementary Figure 2: The relationship between λ, μ, λ^* and μ^* according to Eqn. (3). Estimates are shown for simulations of fossil and phylogenetic data under the birth-death chronospecies model with different proportions of cladogenesis via budding, anagenetic speciation, and extinction without replacement. The proportion of bifurcation events is kept constant ($\beta = 0.5$) to illustrate the effect of variable rates of anagenesis (λ_a). These plots show that even though λ_a and β cannot be estimated directly, $\lambda^* - 2\lambda$ tends to be around 0 when anagenetic speciation and cladogenesis via budding are equally frequent (top row), whereas $\lambda^* - 2\lambda$ tends to be positive when anagenetic speciation is more frequent than budding (bottom row).

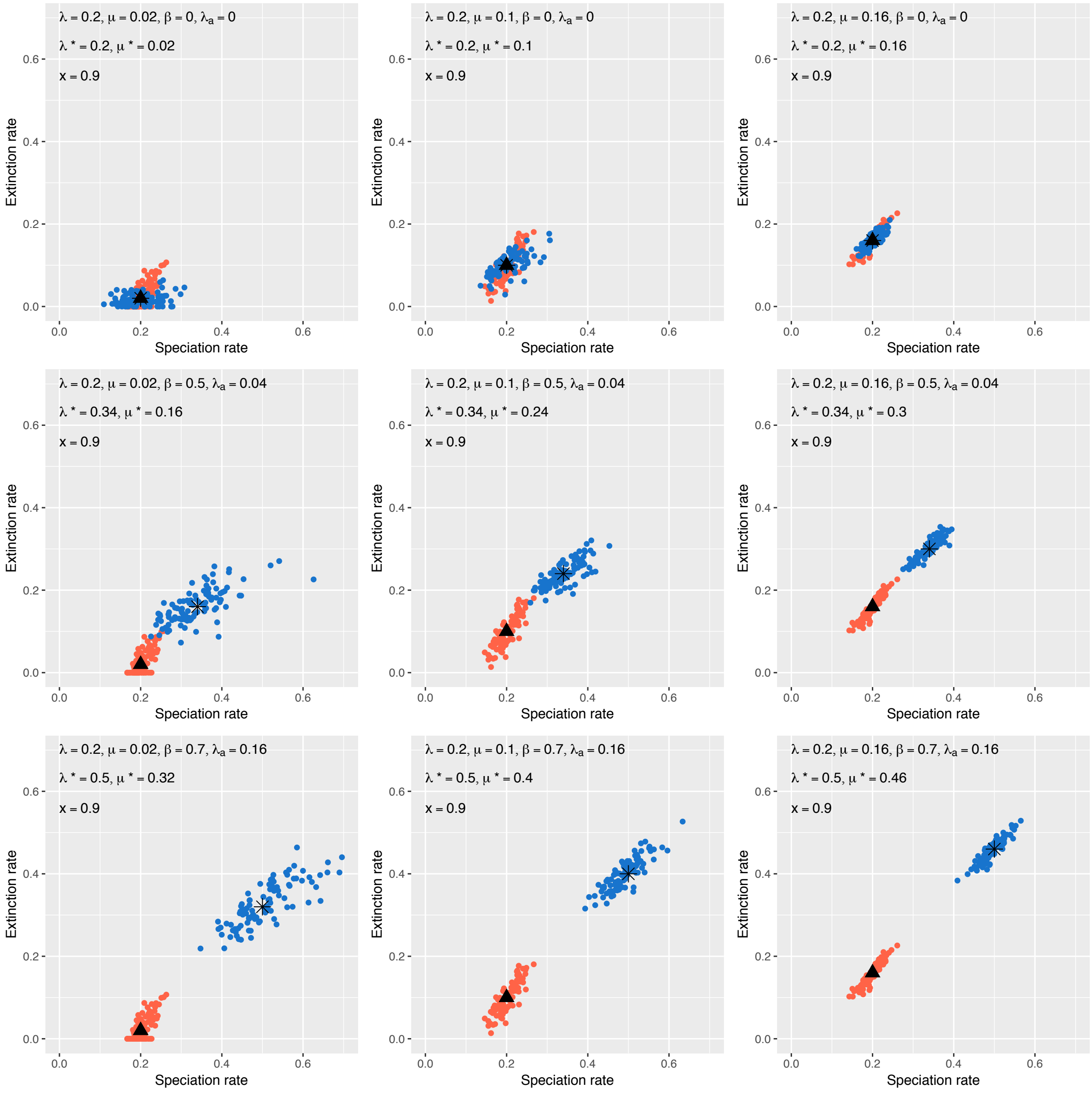

Supplementary Figure 3: Diversification rates estimates for simulations of fossil and phylogenetic data under the birth-death chronospecies model with different proportions of cladogenesis via budding, anagenetic speciation, and extinction without replacement, when 90% of ranges are missing (x). Phylogenetic estimates of the speciation and extinction rates (λ, μ) are shown in red with black triangles representing the true values. The speciation and extinction rates (λ^*, μ^*) estimated from fossil ranges are shown in blue with black stars representing the true values.

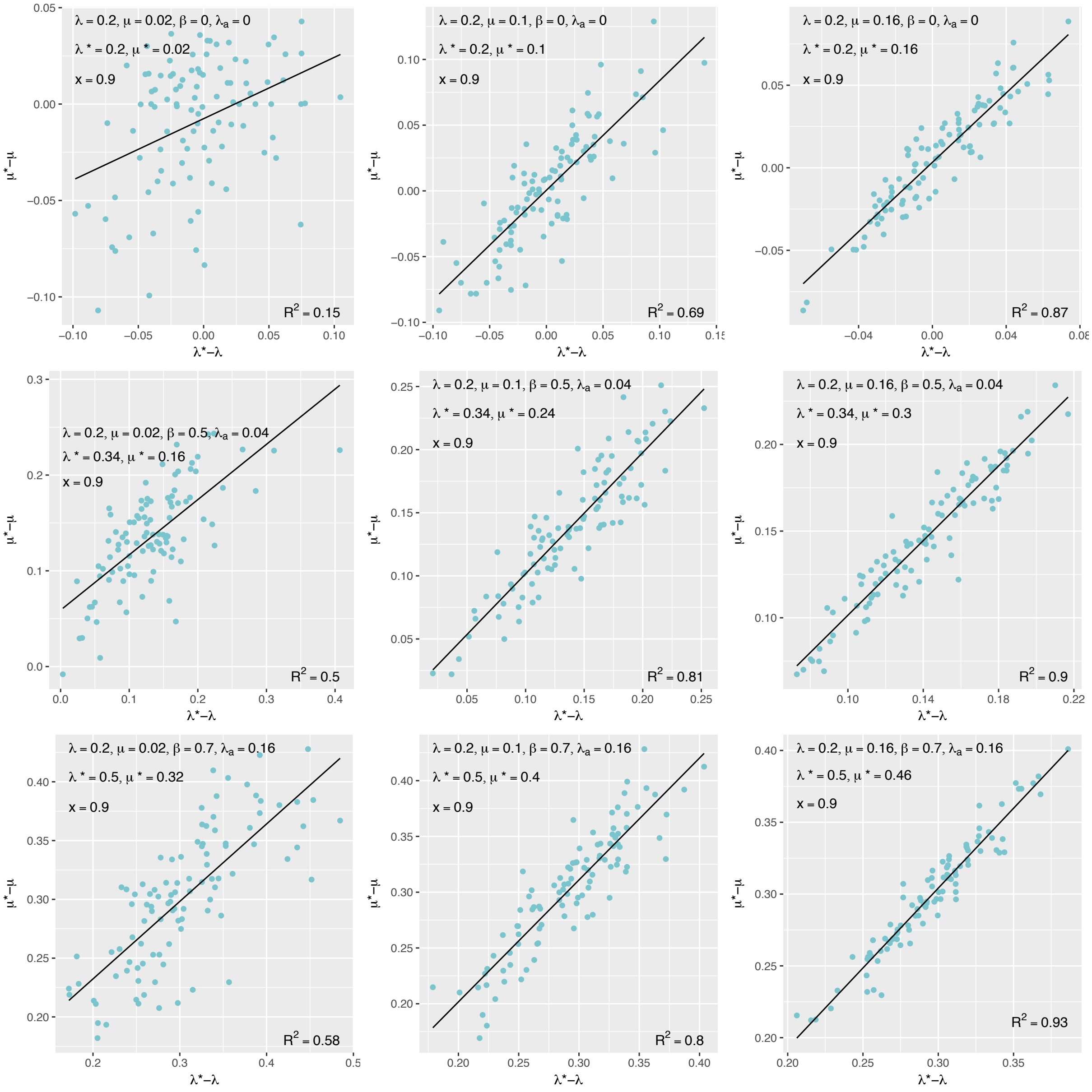

Supplementary Figure 4: The relationship between λ, μ, λ^* and μ^* according to Eqn. (5). Estimates are shown for simulations of fossil and phylogenetic data under the birth-death chronospecies model with different proportions of cladogenesis via budding or bifurcation, anagenetic speciation, and extinction without replacement, when 90% of ranges are randomly removed (x).

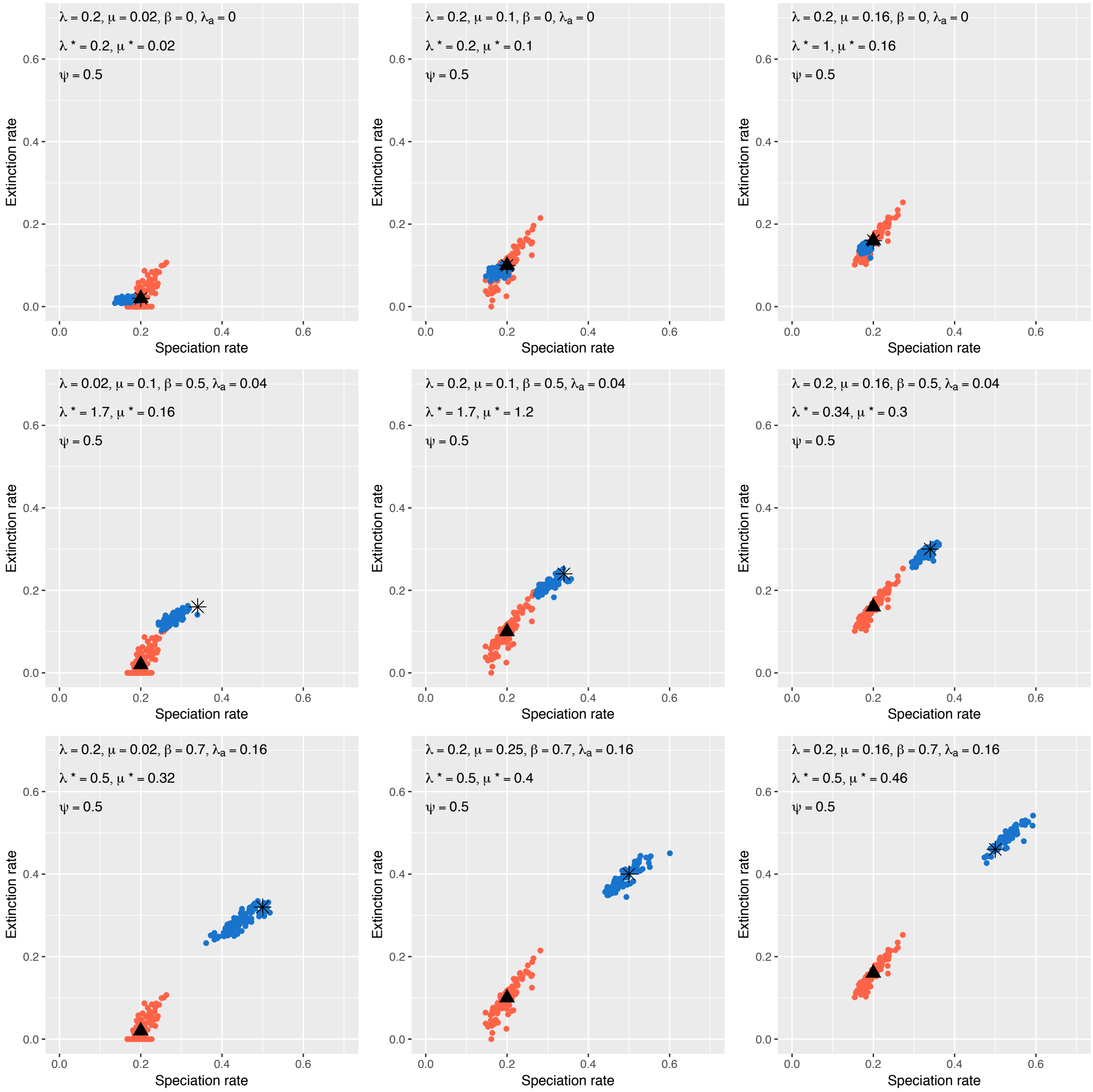

Supplementary Figure 5: Diversification rates estimates for simulations of fossil and phylogenetic data under the birth-death chronospecies model with different proportions of cladogenesis via budding, anagenetic speciation, and extinction without replacement, with Poisson fossil sampling ($\psi = 0.5$). Phylogenetic estimates of the speciation and extinction rates (λ, μ) are shown in red with black triangles representing the true values. The speciation and extinction rates (λ^*, μ^*) estimated from fossil ranges are shown in blue with black stars representing the true values.

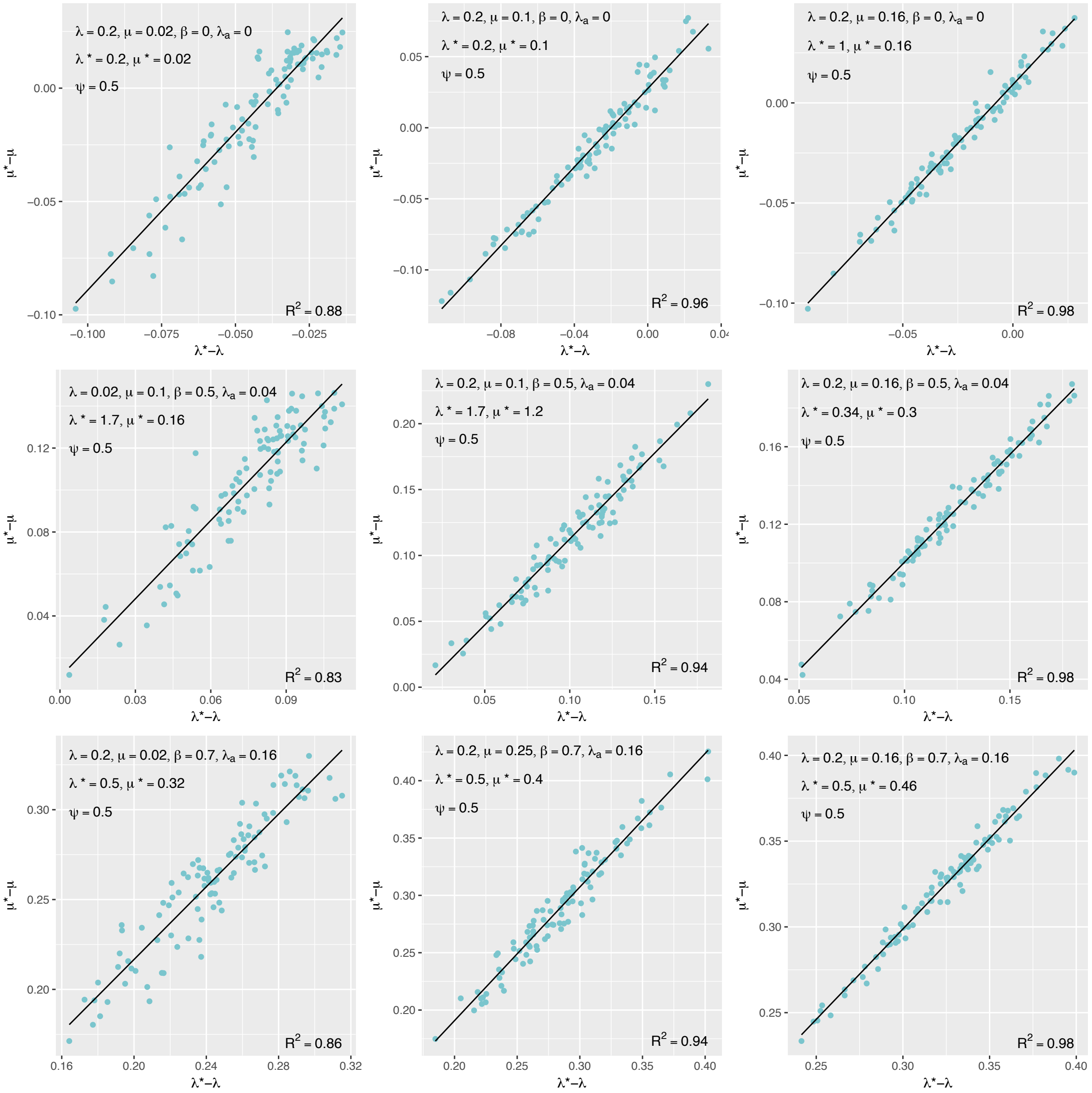

Supplementary Figure 6: The relationship between λ, μ, λ^* and μ^* according to Eqn. (5). Estimates are shown for simulations of fossil and phylogenetic data under the birth-death chronospecies model with different proportions of cladogenesis via budding or bifurcation, anagenetic speciation, and extinction without replacement, with Poisson fossil sampling ($\psi = 0.5$).

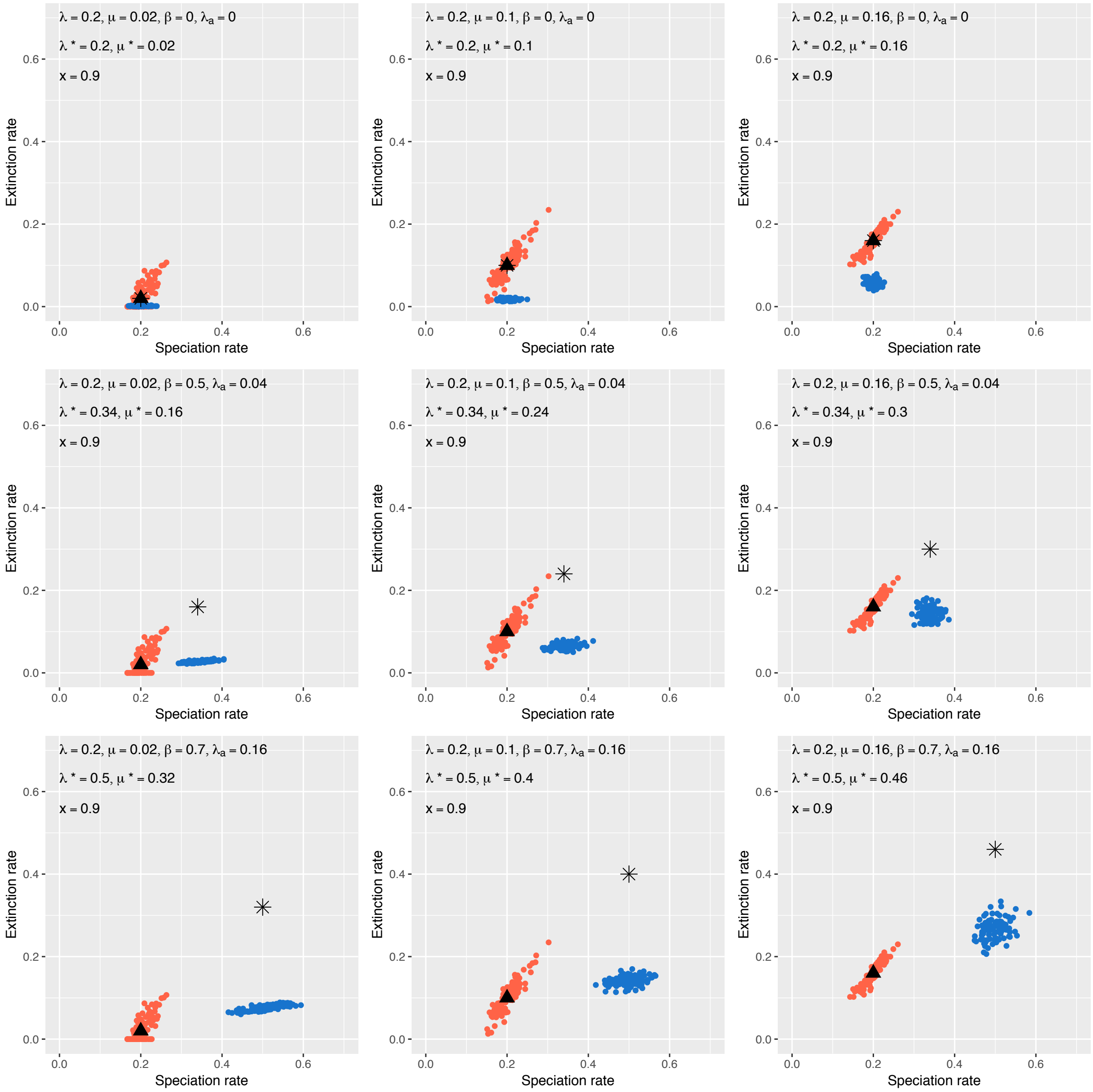

Supplementary Figure 7: Diversification rates estimates for simulations of fossil and phylogenetic data under the birth-death chronospecies model with different proportions of cladogenesis via budding, anagenetic speciation, and extinction without replacement, when 90% of extinct ranges are removed (x). Phylogenetic estimates of the speciation and extinction rates (λ, μ) are shown in red with black triangles representing the true values. The speciation and extinction rates (λ^*, μ^*) estimated from fossil ranges are shown in blue with black stars representing the true values.

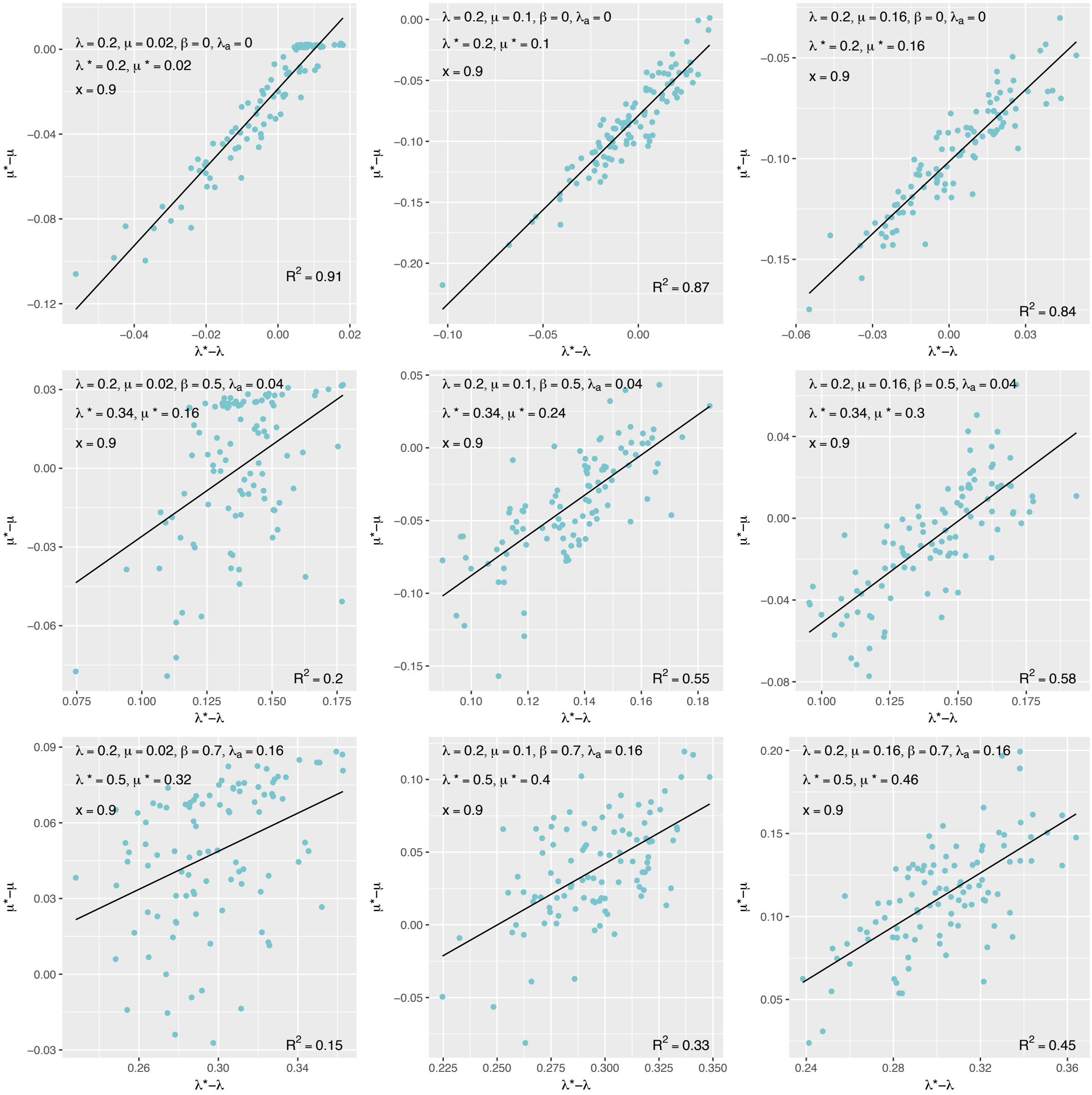

Supplementary Figure 8: The relationship between λ, μ, λ^* and μ^* according to Eqn. (5). Estimates are shown for simulations of fossil and phylogenetic data under the birth-death chronospecies model with different proportions of cladogenesis via budding or bifurcation, anagenetic speciation, and extinction without replacement, when 90% of extinct ranges are removed (x).

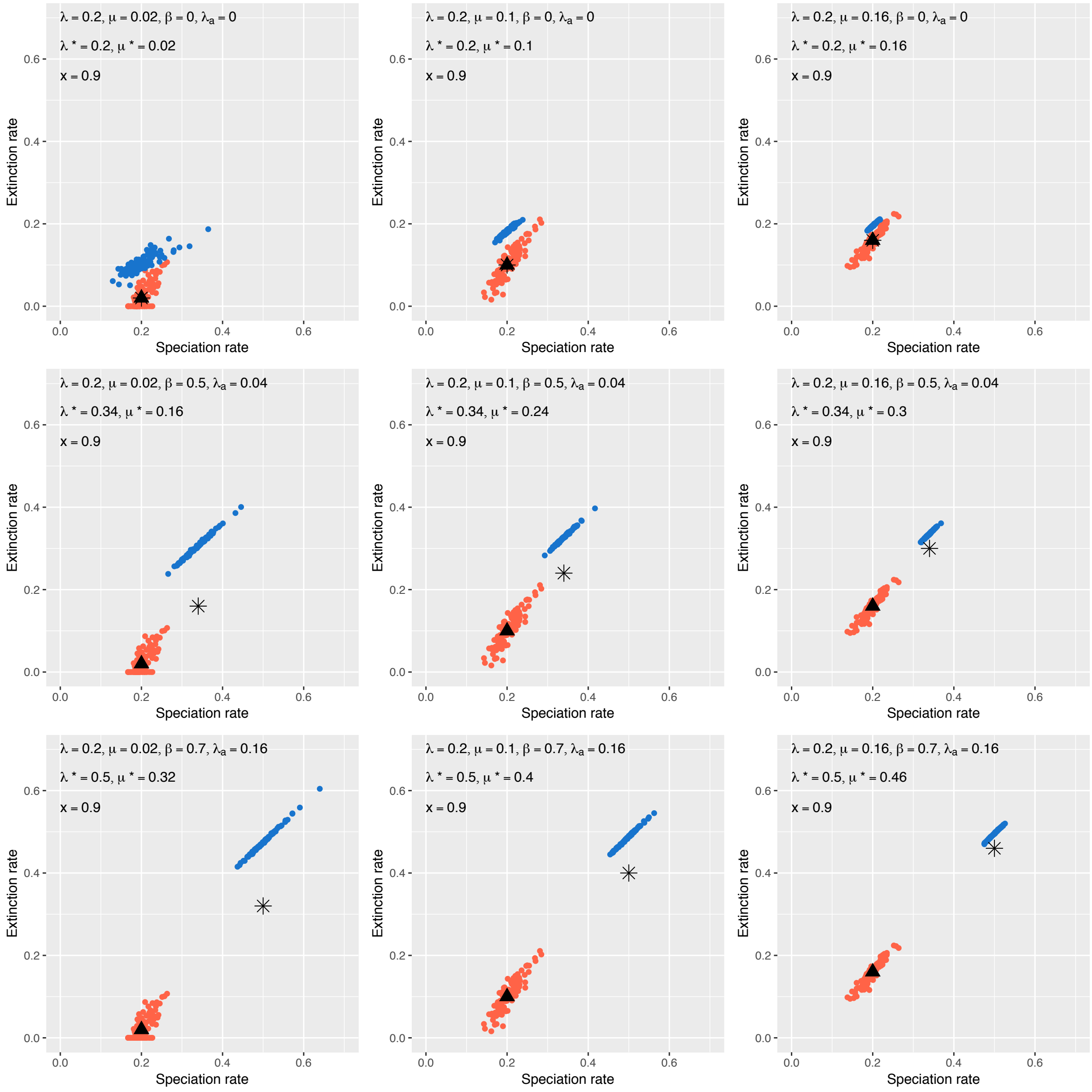

Supplementary Figure 9: Diversification rates estimates for simulations of fossil and phylogenetic data under the birth-death chronospecies model with different proportions of cladogenesis via budding, anagenetic speciation, and extinction without replacement, when 90% of extant ranges are removed (x). Phylogenetic estimates of the speciation and extinction rates (λ, μ) are shown in red with black triangles representing the true values. The speciation and extinction rates (λ^*, μ^*) estimated from fossil ranges are shown in blue with black stars representing the true values.

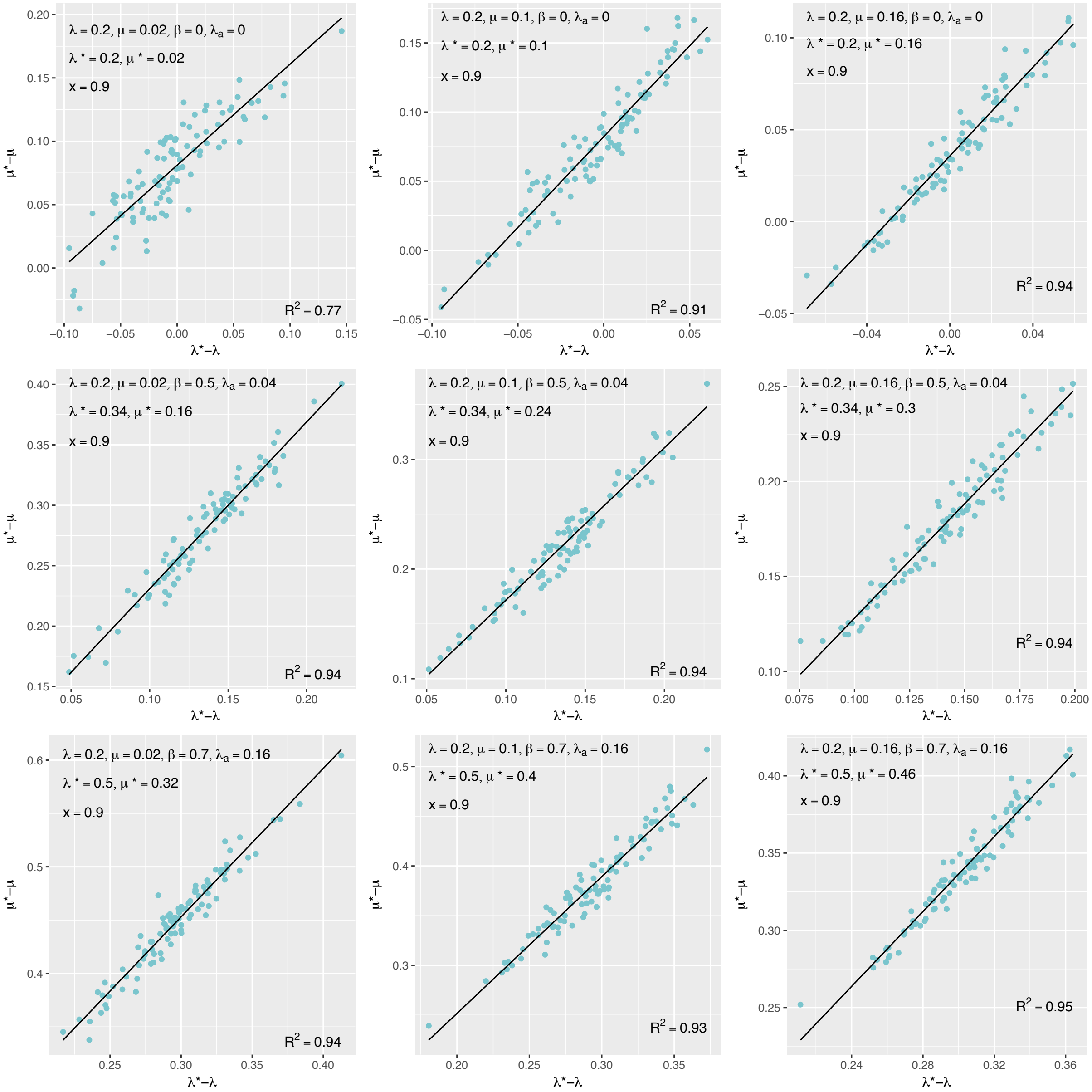

Supplementary Figure 10: The relationship between λ, μ, λ^* and μ^* according to Eqn. (5). Estimates are shown for simulations of fossil and phylogenetic data under the birth-death chronospecies model with different proportions of cladogenesis via budding or bifurcation, anagenetic speciation, and extinction without replacement, when 90% of extant ranges are removed (x).

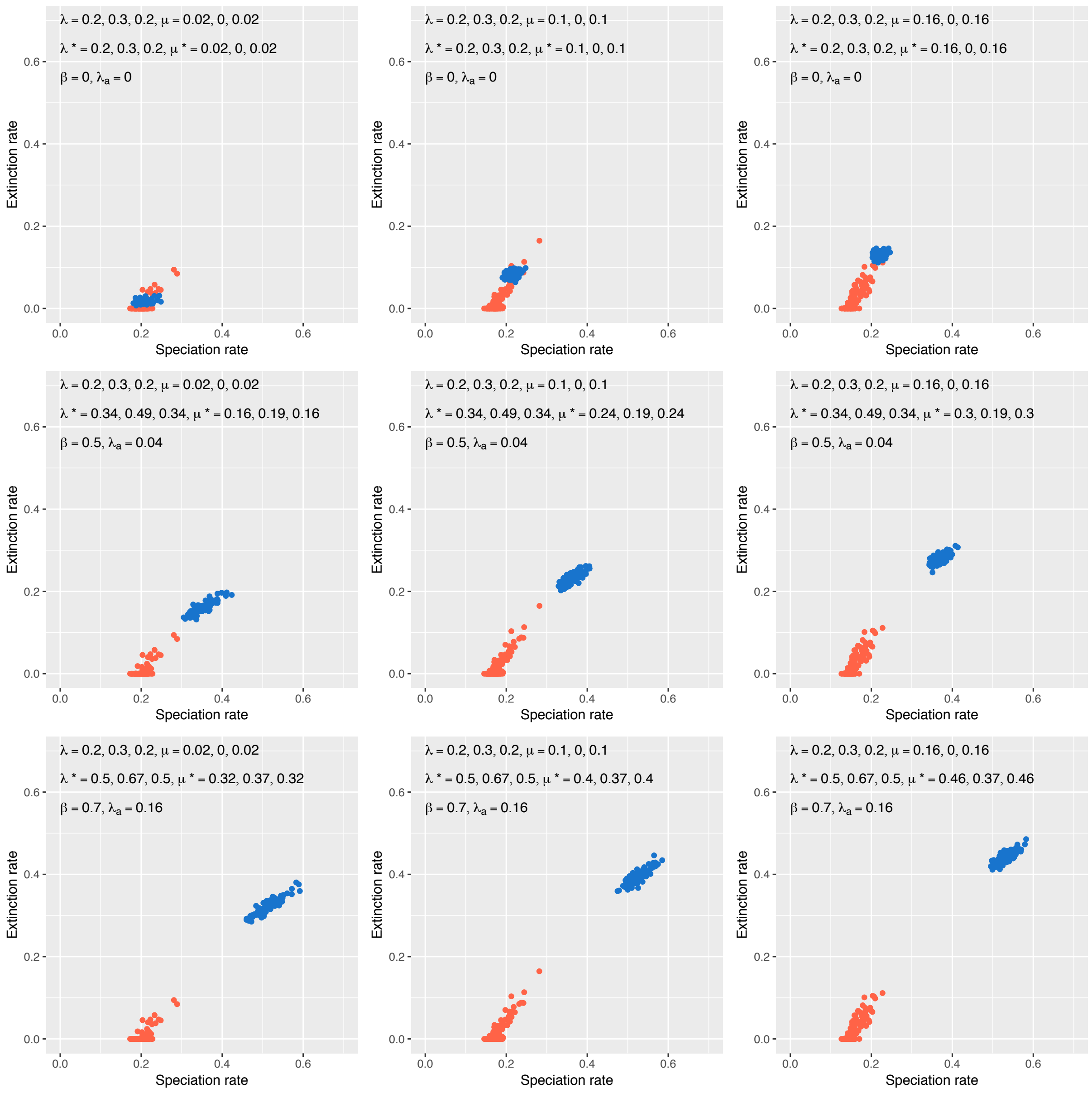

Supplementary Figure 11: Diversification rates estimates for simulations of fossil and phylogenetic data under the birth-death chronospecies model with different proportions of cladogenesis via budding, anagenetic speciation, and extinction without replacement, when diversification rate varies over time. During the period 10-20 myr there is an elevated rate of diversification. Phylogenetic estimates of the speciation and extinction rates (λ, μ) are shown in red. The speciation and extinction rates (λ^*, μ^*) estimated from fossil ranges are shown in blue.

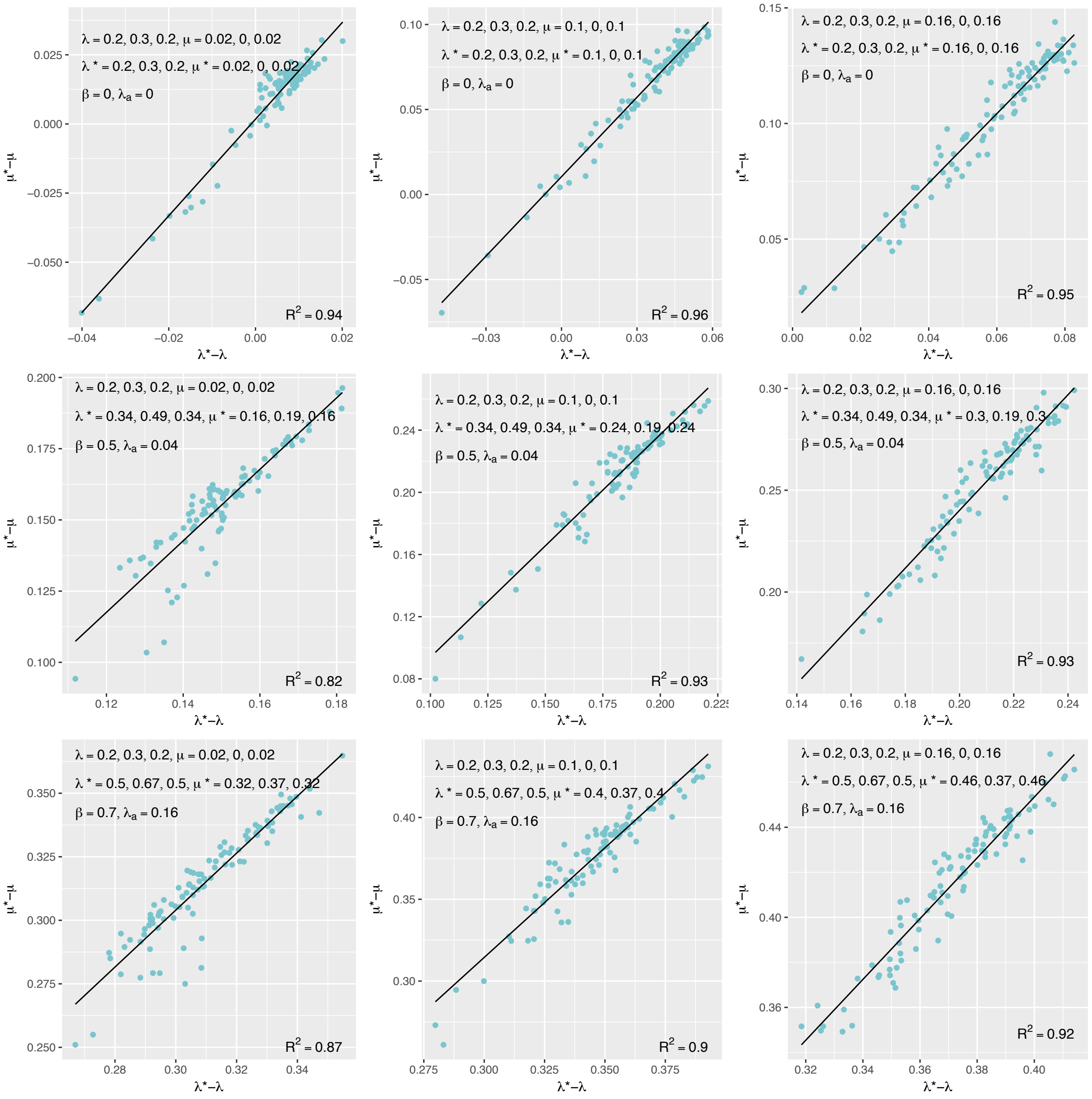

Supplementary Figure 12: The relationship between λ, μ, λ^* and μ^* according to Eqn. (5). Estimates are shown for simulations of fossil and phylogenetic data under the birth-death chronospecies model with different proportions of cladogenesis via budding or bifurcation, anagenetic speciation, and extinction without replacement, when diversification rate varies over time. During the period 10-20 Myr there is an elevated rate of diversification.

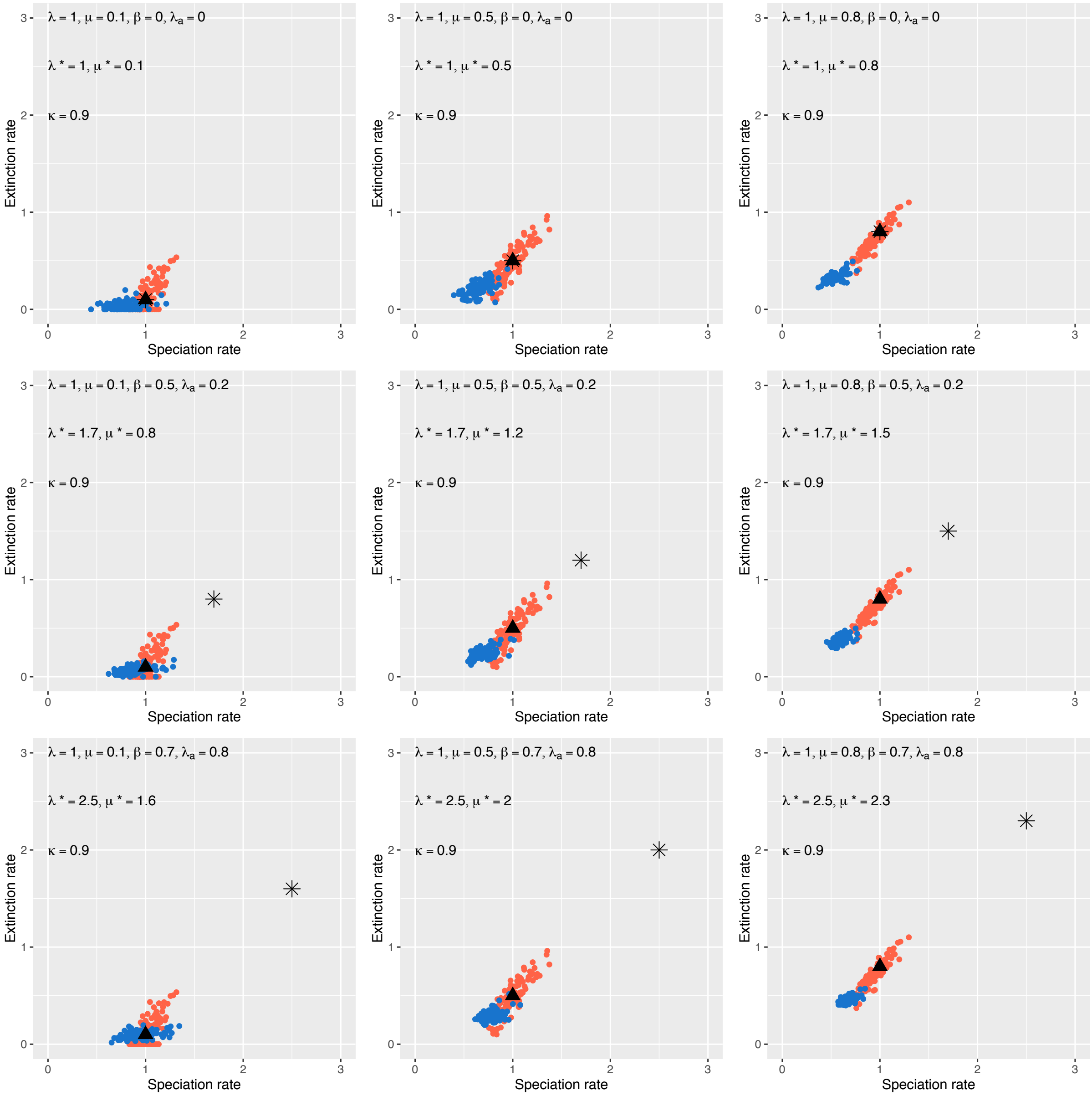

Supplementary Figure 13: Diversification rates estimates for simulations of fossil and phylogenetic data under the birth-death chronospecies model with different proportions of cladogenesis via budding, anagenetic speciation, and extinction without replacement, when 90% of speciation events are cryptic (κ). Phylogenetic estimates of the speciation and extinction rates (λ, μ) are shown in red with black triangles representing the true values. The speciation and extinction rates (λ^*, μ^*) estimated from fossil ranges are shown in blue with black stars representing the true values.

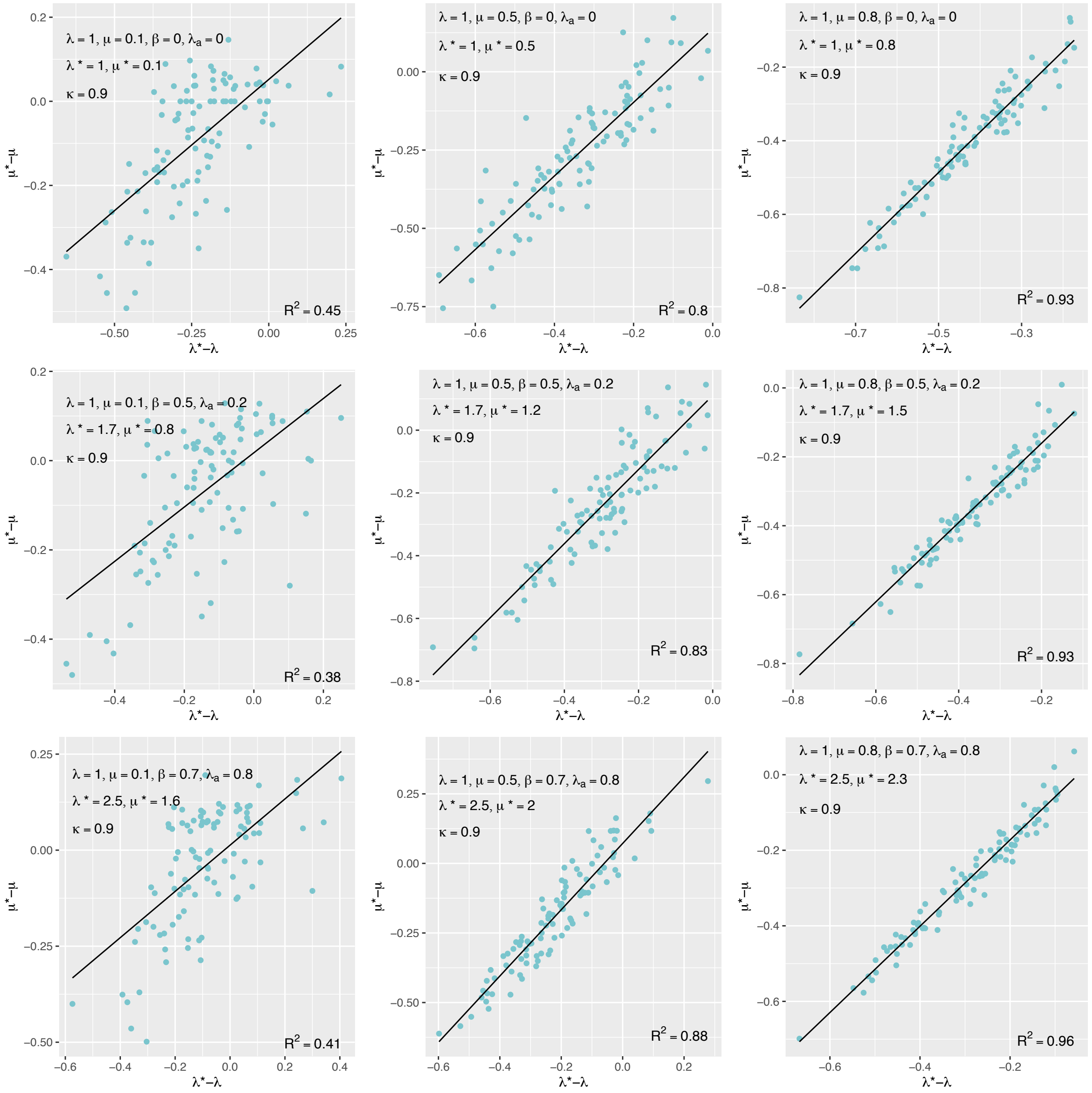

Supplementary Figure 14: The relationship between λ, μ, λ^* and μ^* according to Eqn. (5). Estimates are shown for simulations of fossil and phylogenetic data under the birth-death chronospecies model with different proportions of cladogenesis via budding or bifurcation, anagenetic speciation, and extinction without replacement, when 90% of speciation events are cryptic (κ).

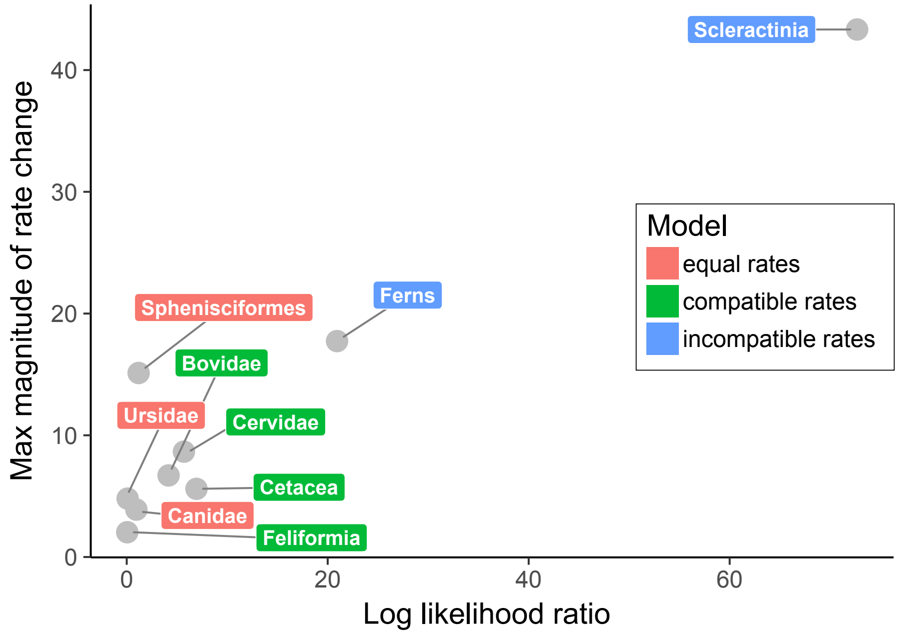

Supplementary Figure 15: Empirical likelihood ratio estimates for the birth-death chronospecies (compatible rates) model versus the incompatible rates model plotted against the amount of rate variation through time estimated from fossil data only. To quantify the amount of rate variation we used the marginal rates inferred by PyRate and calculated the ratio between the highest rates and the lowest. Clade labels are coloured based on the the birth-death model selected by Bayesian analysis (under a constant-rate BDC model), using a threshold of 0.99.

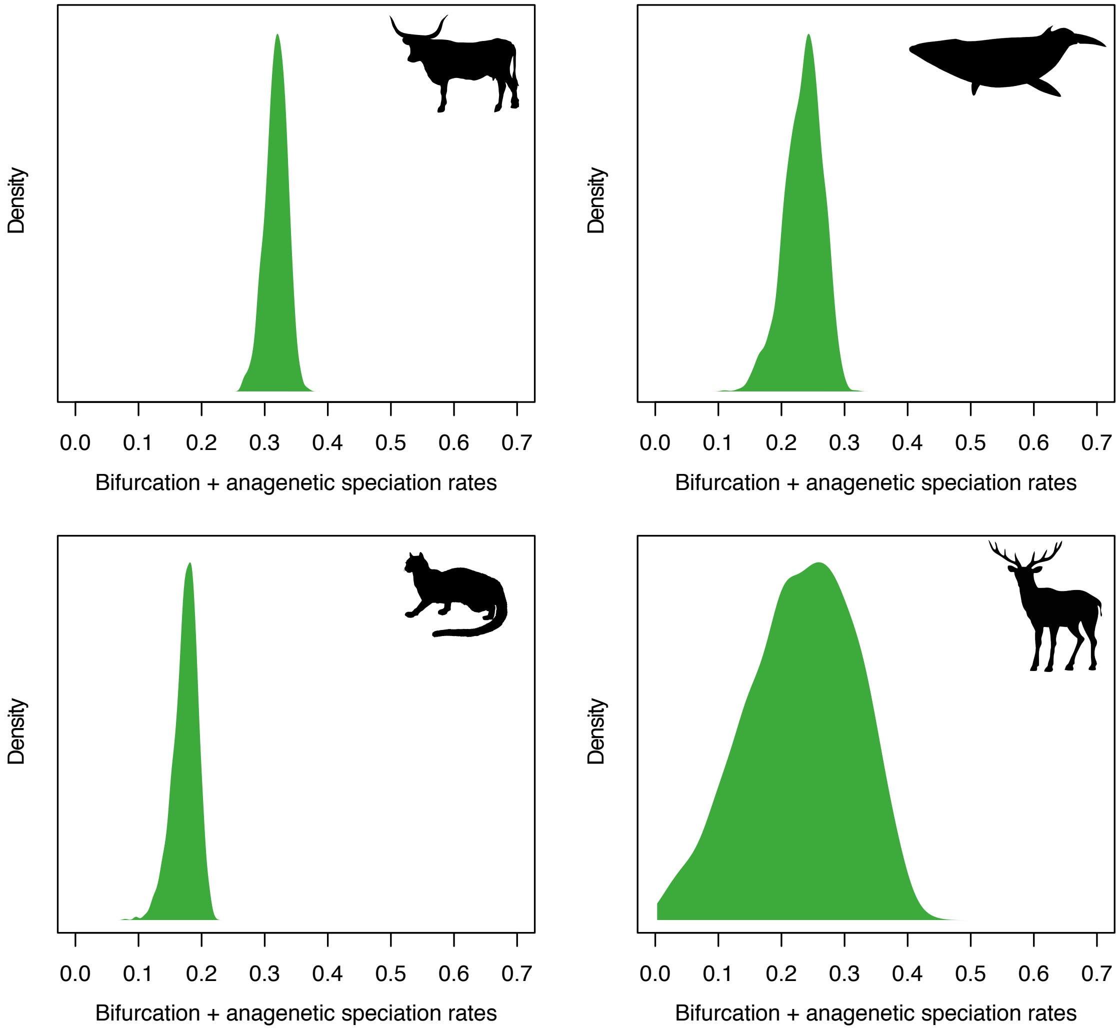

Supplementary Figure 16: Posterior samples of the sum between bifurcating and anagenetic rates of speciation in four mammal clades (Bovidae, Cetacea, Feliformia, Cervidae), as inferred under the birth-death chronospecies model. Although the individual rates cannot be teased apart, their sum is obtained using the properties of the BDC model ($\lambda_a + \lambda\beta = \lambda^* - \lambda$) based on the posterior samples of speciation rates from fossil and phylogenetic data (λ^*, and λ, respectively).

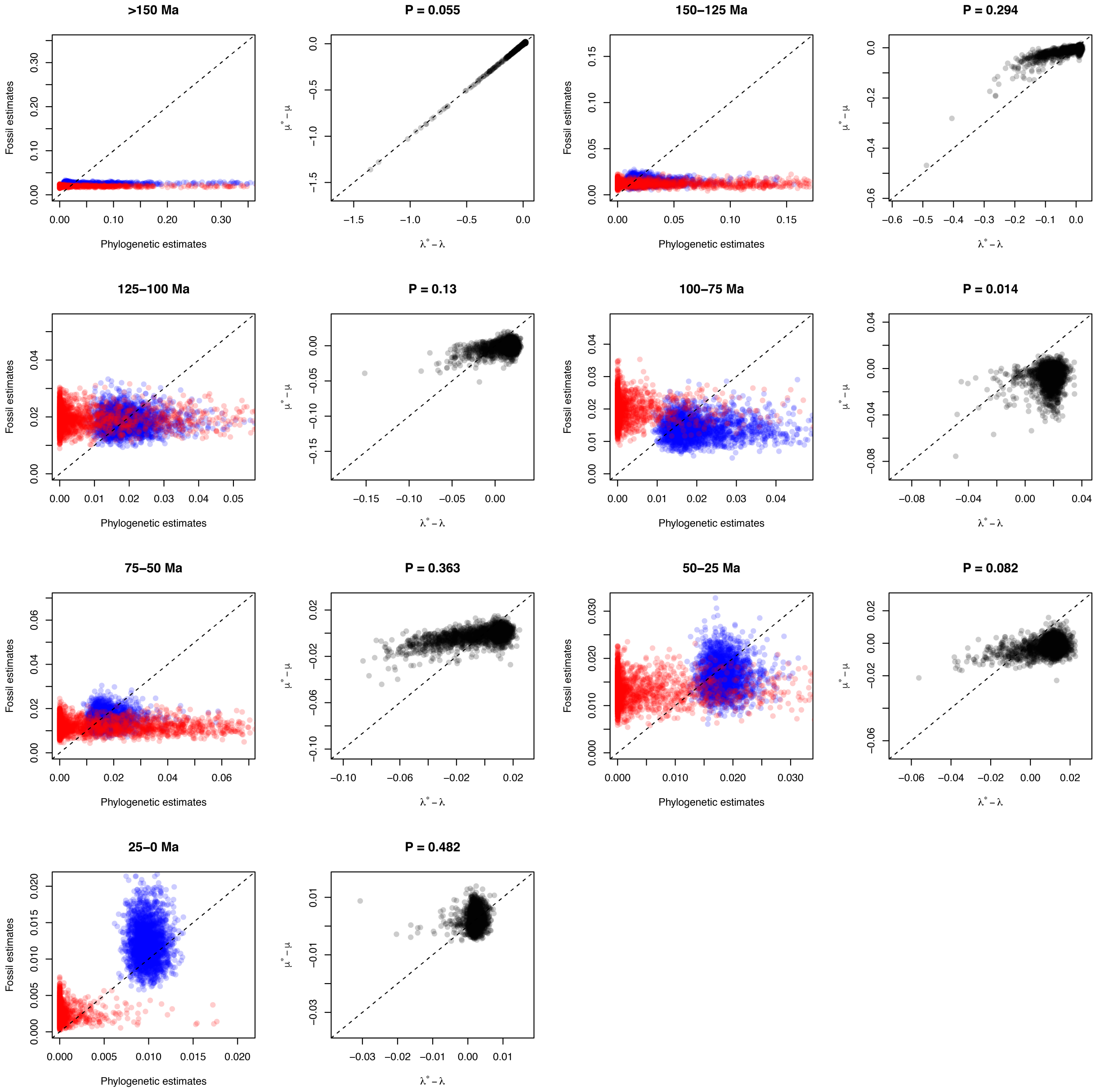

Supplementary Figure 17: Results from a joint Bayesian analysis of fossil and phylogenetic data for ferns under the skyline model. Posterior samples of origination rates (in blue) and extinction rates (in red) jointly inferred from the two data types are plotted against one another; posterior samples of the two terms of equation (5) are shown in black. Six rate shifts were used to account for rate heterogeneity. Under this model, the fern phylogenetic and fossil data conform to the BDC model, which was instead rejected under the assumption of constant rates (Fig. 3).

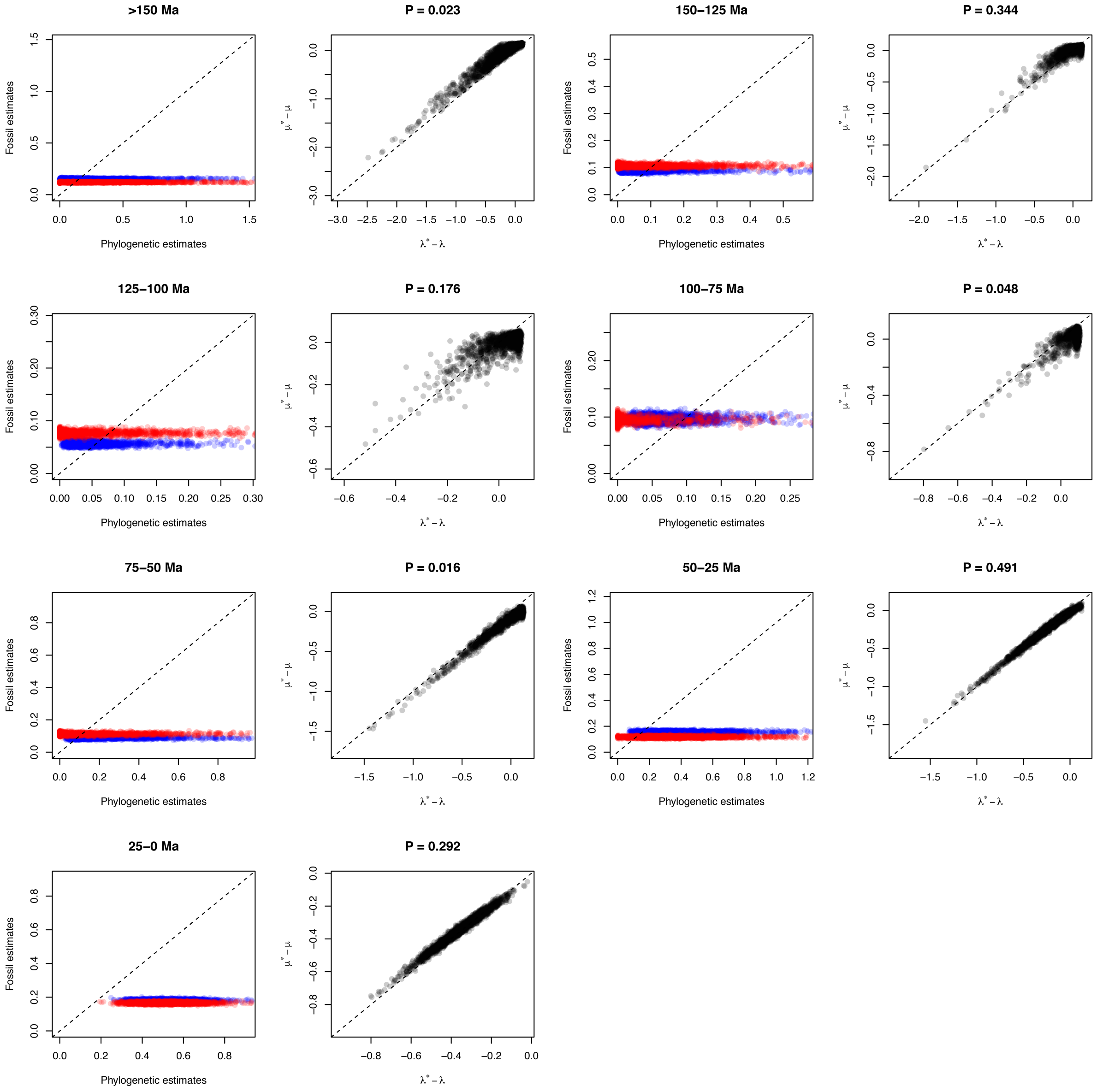

Supplementary Figure 18: Results from a joint Bayesian analysis of fossil and phylogenetic data for corals under the skyline model. Posterior samples of speciation rates (in blue) and extinction rates (in red) jointly inferred from the two data types are plotted against one another; posterior samples of the two terms of equation (5) are shown in black. Six rate shifts were used to account for rate heterogeneity. Under this model, the coral phylogenetic and fossil data rejects to the birth-death chronospecies model, as in the case of constant rates. There is little phylogenetic information in all estimates prior to 25 Ma (as shown by the large spread of posterior values) and in the phylogenetic rates significantly exceed fossil rates between 25 and 0 Ma, thus contradicting the expectations of the BDC model.

Supplementary tables

Supplementary Table 1: Speciation and extinction rates estimated from simulated datasets of extant phylogenies and stratigraphic ranges under the equal, compatible and incompatible rates models. Rate parameters are averaged over 100 simulations under each parameter setting.

True parameter values					Equal rates			Compatible rates					Incompatible rates						Model comparison†					
																			Eq. v Co.		Eq. v In.		Co. v In.	
λ	μ	λ^*	μ^*	$d = d^*$	$\hat{\lambda} = \hat{\lambda}^*$	$\hat{\mu} = \hat{\mu}^*$	$\hat{d} = \hat{d}^*$	$\hat{\lambda}$	$\hat{\mu}$	$\hat{\lambda}^*$	$\hat{\mu}^*$	$\hat{d} = \hat{d}^*$	$\hat{\lambda}$	$\hat{\mu}$	$\hat{\lambda}^*$	$\hat{\mu}^*$	$\hat{d}$	$\hat{d}^*$	.95	.99	.95	.99	.95	.99
$\beta = 0, \lambda_a = 0$																								
0.2	0.02	0.2	0.02	0.18	0.2	0.02	0.18	0.2	0.01	0.2	0.02	0.18	0.2	0.03	0.2	0.02	0.18	0.18	1	1	1	1	1	1
0.2	0.1	0.2	0.1	0.1	0.2	0.1	0.1	0.19	0.09	0.2	0.1	0.1	0.2	0.11	0.2	0.1	0.1	0.1	1	1	0.99	1	0.96	1
0.2	0.16	0.2	0.16	0.04	0.2	0.16	0.04	0.19	0.15	0.2	0.16	0.04	0.2	0.15	0.2	0.16	0.04	0.04	0.96	1	0.99	1	0.99	0.99
$\beta = 0.5, \lambda_a = 0.04$																								
0.2	0.02	0.34	0.16	0.18	0.31	0.16	0.15	0.2	0.02	0.34	0.16	0.18	0.2	0.03	0.34	0.16	0.18	0.18	0	0	0	0.15	1	1
0.2	0.1	0.34	0.24	0.1	0.32	0.24	0.08	0.2	0.1	0.34	0.24	0.1	0.2	0.11	0.34	0.24	0.1	0.1	0	0.06	0.05	0.19	1	1
0.2	0.16	0.34	0.3	0.04	0.33	0.3	0.03	0.2	0.16	0.34	0.3	0.04	0.2	0.15	0.34	0.3	0.04	0.04	0	0.01	0.01	0.07	1	1
$\beta = 0.7, \lambda_a = 0.16$																								
0.2	0.02	0.5	0.32	0.18	0.45	0.33	0.12	0.2	0.02	0.5	0.32	0.18	0.2	0.03	0.5	0.32	0.18	0.18	0	0	0	0	1	1
0.2	0.1	0.5	0.4	0.1	0.47	0.4	0.06	0.2	0.1	0.5	0.4	0.1	0.2	0.11	0.5	0.4	0.1	0.1	0	0	0	0	1	1
0.2	0.16	0.5	0.46	0.04	0.49	0.46	0.02	0.2	0.15	0.5	0.46	0.04	0.2	0.15	0.5	0.46	0.04	0.04	0	0	0	0	1	1

†Values shown in this section are the proportion of the simulated replicates for which the null model cannot be rejected, given the level of significance (0.95 or 0.99).

Supplementary Table 2: Speciation and extinction rates estimated from simulated data under the equal, compatible and incompatible rates models. The proportion of bifurcation events is kept constant ($\beta = 0.5$) to illustrate the impact of variable rates of anagenesis (λ_a). Estimated parameters are averaged over 100 simulations under each parameter setting.

																			Model comparison†					
True parameter values					Equal rates			Compatible rates					Incompatible rates						Eq. v Co.		Eq. v In.		Co. v In.	
λ	μ	λ^*	μ^*	$d = d^*$	$\hat{\lambda} = \hat{\lambda}^*$	$\hat{\mu} = \hat{\mu}^*$	$\hat{d} = \hat{d}^*$	$\hat{\lambda}$	$\hat{\mu}$	$\hat{\lambda}^*$	$\hat{\mu}^*$	$\hat{d} = \hat{d}^*$	$\hat{\lambda}$	$\hat{\mu}$	$\hat{\lambda}^*$	$\hat{\mu}^*$	$\hat{d}$	$\hat{d}^*$	.95	.99	.95	.99	.95	.99
$\beta = 0.5, \lambda_a = 0.1$																								
0.2	0.02	0.4	0.22	0.18	0.36	0.22	0.14	0.2	0.02	0.4	0.22	0.18	0.2	0.03	0.4	0.22	0.18	0.18	0	0	0	0	1	1
0.2	0.1	0.4	0.3	0.1	0.38	0.3	0.07	0.2	0.1	0.4	0.3	0.1	0.2	0.1	0.4	0.3	0.1	0.1	0	0	0	0.02	1	1
0.2	0.16	0.4	0.36	0.04	0.39	0.36	0.03	0.19	0.15	0.4	0.36	0.04	0.19	0.15	0.4	0.36	0.05	0.04	0	0	0	0	1	1
$\beta = 0.5, \lambda_a = 0.2$																								
0.2	0.02	0.5	0.32	0.18	0.45	0.32	0.13	0.2	0.02	0.5	0.32	0.18	0.2	0.03	0.5	0.32	0.18	0.18	0	0	0	0	1	1
0.2	0.1	0.5	0.4	0.1	0.47	0.4	0.06	0.2	0.1	0.5	0.4	0.1	0.2	0.1	0.5	0.4	0.1	0.1	0	0	0	0	1	1
0.2	0.16	0.5	0.46	0.04	0.49	0.46	0.03	0.19	0.15	0.5	0.46	0.04	0.19	0.15	0.5	0.46	0.05	0.04	0	0	0	0	1	1
$\beta = 0.5, \lambda_a = 0.3$																								
0.2	0.02	0.6	0.42	0.18	0.54	0.43	0.11	0.2	0.02	0.6	0.42	0.18	0.2	0.03	0.6	0.42	0.18	0.18	0	0	0	0	1	1
0.2	0.1	0.6	0.5	0.1	0.56	0.5	0.06	0.2	0.1	0.6	0.5	0.1	0.2	0.1	0.6	0.5	0.1	0.1	0	0	0	0	1	1
0.2	0.16	0.6	0.56	0.04	0.58	0.56	0.02	0.19	0.15	0.6	0.56	0.04	0.19	0.15	0.6	0.56	0.05	0.04	0	0	0	0	1	1

†Values shown in this section are the proportion of the simulated replicates for which the null model cannot be rejected, given the level of significance (0.95 or 0.99).

Supplementary Table 3: Speciation and extinction rates estimated from simulated data under the equal, compatible and incompatible rates models when a proportion (x) of ranges are uniformly removed. Estimated parameters are averaged over 100 simulations under each parameter setting.

True parameter values					Equal rates			Compatible rates					Incompatible rates						Model comparison†					
																			Eq. v Co.		Eq. v In.		Co. v In.	
λ	μ	λ^*	μ^*	$d = d^*$	$\hat{\lambda} = \hat{\lambda}^*$	$\hat{\mu} = \hat{\mu}^*$	$\hat{d} = \hat{d}^*$	$\hat{\lambda}$	$\hat{\mu}$	$\hat{\lambda}^*$	$\hat{\mu}^*$	$\hat{d} = \hat{d}^*$	$\hat{\lambda}$	$\hat{\mu}$	$\hat{\lambda}^*$	$\hat{\mu}^*$	$\hat{d}$	$\hat{d}^*$	.95	.99	.95	.99	.95	.99
**Proportion of missing ranges $x = 0.1$**																								
$\beta = 0, \lambda_a = 0$																								
0.2	0.02	0.2	0.02	0.18	0.2	0.02	0.18	0.2	0.01	0.2	0.02	0.18	0.2	0.03	0.2	0.02	0.18	0.18	1	1	1	1	1	1
0.2	0.1	0.2	0.1	0.1	0.2	0.1	0.1	0.19	0.09	0.2	0.1	0.1	0.2	0.1	0.2	0.1	0.1	0.1	0.97	0.99	0.98	1	0.98	1
0.2	0.16	0.2	0.16	0.04	0.2	0.16	0.04	0.19	0.15	0.2	0.16	0.04	0.19	0.15	0.2	0.16	0.04	0.04	0.95	1	1	1	1	1
$\beta = 0.5, \lambda_a = 0.04$																								
0.2	0.02	0.34	0.16	0.18	0.31	0.16	0.15	0.2	0.02	0.34	0.16	0.18	0.2	0.03	0.34	0.16	0.18	0.18	0	0.02	0.03	0.2	1	1
0.2	0.1	0.34	0.24	0.1	0.32	0.24	0.08	0.2	0.1	0.34	0.24	0.1	0.2	0.1	0.34	0.24	0.1	0.1	0	0.03	0.03	0.15	1	1
0.2	0.16	0.34	0.3	0.04	0.33	0.3	0.03	0.2	0.15	0.34	0.3	0.04	0.19	0.15	0.34	0.3	0.04	0.04	0	0	0	0.05	1	1
$\beta = 0.7, \lambda_a = 0.16$																								
0.2	0.02	0.5	0.32	0.18	0.45	0.33	0.12	0.2	0.02	0.5	0.32	0.18	0.2	0.03	0.5	0.32	0.18	0.18	0	0	0	0	1	1
0.2	0.1	0.5	0.4	0.1	0.47	0.4	0.06	0.2	0.1	0.5	0.4	0.1	0.2	0.1	0.5	0.4	0.1	0.1	0	0	0	0	1	1
0.2	0.16	0.5	0.46	0.04	0.49	0.46	0.02	0.2	0.15	0.5	0.46	0.04	0.19	0.15	0.5	0.46	0.04	0.04	0	0	0	0	1	1
**Proportion of missing ranges $x = 0.5$**																								
$\beta = 0, \lambda_a = 0$																								
0.2	0.02	0.2	0.02	0.18	0.2	0.02	0.18	0.2	0.01	0.2	0.02	0.18	0.2	0.03	0.2	0.02	0.18	0.18	1	1	1	1	1	1
0.2	0.1	0.2	0.1	0.1	0.2	0.1	0.1	0.19	0.09	0.2	0.1	0.1	0.2	0.1	0.2	0.1	0.1	0.1	0.96	0.99	0.99	1	0.97	1
0.2	0.16	0.2	0.16	0.04	0.2	0.16	0.04	0.19	0.15	0.2	0.16	0.04	0.2	0.15	0.2	0.16	0.04	0.04	0.91	1	1	1	0.99	1
$\beta = 0.5, \lambda_a = 0.04$																								
0.2	0.02	0.34	0.16	0.18	0.3	0.16	0.14	0.2	0.02	0.34	0.16	0.18	0.2	0.03	0.34	0.16	0.18	0.18	0	0.11	0.11	0.31	1	1
0.2	0.1	0.34	0.24	0.1	0.31	0.24	0.08	0.2	0.1	0.34	0.24	0.1	0.2	0.1	0.34	0.24	0.1	0.1	0	0.08	0.09	0.22	1	1
0.2	0.16	0.34	0.3	0.04	0.33	0.3	0.03	0.2	0.15	0.34	0.3	0.04	0.2	0.15	0.34	0.3	0.04	0.04	0	0	0	0.07	1	1
$\beta = 0.7, \lambda_a = 0.16$																								
0.2	0.02	0.5	0.32	0.18	0.43	0.32	0.11	0.2	0.03	0.5	0.32	0.18	0.2	0.03	0.5	0.32	0.18	0.18	0	0	0	0	1	1
0.2	0.1	0.5	0.4	0.1	0.45	0.4	0.06	0.2	0.1	0.5	0.4	0.1	0.2	0.1	0.5	0.4	0.1	0.1	0	0	0	0	1	1
0.2	0.16	0.5	0.46	0.04	0.48	0.46	0.02	0.2	0.15	0.5	0.46	0.04	0.2	0.15	0.5	0.46	0.04	0.04	0	0	0	0	1	1

– table continued on the next page

Table continued from the previous page.

																			Model comparison†					
True parameter values					Equal rates			Compatible rates					Incompatible rates						Eq. v Co.		Eq. v In.		Co. v In.	
λ	μ	λ^*	μ^*	$d = d^*$	$\hat{\lambda} = \hat{\lambda}^*$	$\hat{\mu} = \hat{\mu}^*$	$\hat{d} = \hat{d}^*$	$\hat{\lambda}$	$\hat{\mu}$	$\hat{\lambda}^*$	$\hat{\mu}^*$	$\hat{d} = \hat{d}^*$	$\hat{\lambda}$	$\hat{\mu}$	$\hat{\lambda}^*$	$\hat{\mu}^*$	$\hat{d}$	$\hat{d}^*$	.95	.99	.95	.99	.95	.99
**Proportion of missing ranges $x = 0.9$**																								
$\beta = 0, \lambda_a = 0$																								
0.2	0.02	0.2	0.02	0.18	0.2	0.02	0.18	0.2	0.01	0.2	0.02	0.18	0.2	0.03	0.2	0.02	0.18	0.18	1	1	0.99	1	0.92	1
0.2	0.1	0.2	0.1	0.1	0.2	0.1	0.1	0.19	0.09	0.21	0.11	0.1	0.2	0.1	0.2	0.1	0.1	0.1	0.92	0.97	0.97	1	0.95	1
0.2	0.16	0.2	0.16	0.04	0.2	0.16	0.04	0.19	0.15	0.2	0.16	0.04	0.2	0.16	0.2	0.16	0.04	0.04	0.88	0.99	0.98	1	0.98	1
$\beta = 0.5, \lambda_a = 0.04$																								
0.2	0.02	0.34	0.16	0.18	0.27	0.13	0.14	0.2	0.03	0.34	0.16	0.18	0.2	0.03	0.34	0.16	0.18	0.18	0.11	0.35	0.37	0.65	1	1
0.2	0.1	0.34	0.24	0.1	0.28	0.21	0.07	0.2	0.1	0.34	0.24	0.1	0.2	0.1	0.34	0.24	0.1	0.1	0.07	0.23	0.26	0.39	1	1
0.2	0.16	0.34	0.3	0.04	0.31	0.28	0.03	0.2	0.16	0.34	0.3	0.04	0.2	0.16	0.34	0.3	0.04	0.04	0	0.07	0.08	0.24	1	1
$\beta = 0.7, \lambda_a = 0.16$																								
0.2	0.02	0.5	0.32	0.18	0.36	0.25	0.11	0.2	0.03	0.51	0.33	0.18	0.2	0.03	0.51	0.33	0.18	0.18	0	0	0	0	1	1
0.2	0.1	0.5	0.4	0.1	0.39	0.33	0.06	0.2	0.1	0.5	0.4	0.1	0.2	0.1	0.49	0.4	0.1	0.09	0	0	0	0	1	1
0.2	0.16	0.5	0.46	0.04	0.44	0.42	0.02	0.2	0.16	0.49	0.45	0.04	0.2	0.16	0.49	0.46	0.04	0.04	0	0	0	0	1	1

†Values shown in this section are the proportion of the simulated replicates for which the null model cannot be rejected, given the level of significance (0.95 or 0.99).

Supplementary Table 4: Speciation and extinction rates estimated from simulated data under the equal, compatible and incompatible rates models when fossils (and therefore ranges) are sampled under a Poisson sampling process ($\psi = 0.5$). Estimated parameters are averaged over 100 simulations under each parameter setting.

																			Model comparison†					
True parameter values					Equal rates			Compatible rates					Incompatible rates						Eq. v Co.		Eq. v In.		Co. v In.	
λ	μ	λ^*	μ^*	$d = d^*$	$\hat{\lambda} = \hat{\lambda}^*$	$\hat{\mu} = \hat{\mu}^*$	$\hat{d} = \hat{d}^*$	$\hat{\lambda}$	$\hat{\mu}$	$\hat{\lambda}^*$	$\hat{\mu}^*$	$\hat{d} = \hat{d}^*$	$\hat{\lambda}$	$\hat{\mu}$	$\hat{\lambda}^*$	$\hat{\mu}^*$	$\hat{d}$	$\hat{d}^*$	.95	.99	.95	.99	.95	.99
$\beta = 0, \lambda_a = 0$																								
0.2	0.02	0.2	0.02	0.18	0.18	0.02	0.17	0.18	0.02	0.18	0.02	0.17	0.2	0.03	0.16	0.02	0.18	0.15	1	1	0.81	0.98	0.35	0.88
0.2	0.1	0.2	0.1	0.1	0.18	0.08	0.1	0.18	0.08	0.18	0.08	0.1	0.2	0.1	0.17	0.08	0.1	0.09	1	1	0.87	0.97	0.73	0.91
0.2	0.16	0.2	0.16	0.04	0.18	0.14	0.04	0.18	0.14	0.18	0.14	0.04	0.2	0.16	0.18	0.14	0.04	0.04	1	1	0.97	0.99	0.88	0.98
$\beta = 0.5, \lambda_a = 0.04$																								
0.2	0.02	0.34	0.16	0.18	0.27	0.13	0.14	0.21	0.05	0.29	0.13	0.16	0.2	0.03	0.28	0.13	0.18	0.15	0.19	0.61	0.54	0.83	1	1
0.2	0.1	0.34	0.24	0.1	0.29	0.21	0.08	0.2	0.11	0.31	0.21	0.1	0.2	0.1	0.3	0.21	0.1	0.09	0.08	0.28	0.3	0.49	1	1
0.2	0.16	0.34	0.3	0.04	0.32	0.29	0.03	0.2	0.16	0.33	0.29	0.04	0.2	0.16	0.33	0.29	0.04	0.04	0.02	0.08	0.09	0.26	1	1
$\beta = 0.7, \lambda_a = 0.16$																								
0.2	0.02	0.5	0.32	0.18	0.4	0.28	0.12	0.21	0.03	0.45	0.28	0.17	0.2	0.03	0.45	0.29	0.18	0.16	0	0	0	0	1	1
0.2	0.1	0.5	0.4	0.1	0.44	0.38	0.06	0.2	0.1	0.49	0.39	0.1	0.2	0.1	0.49	0.39	0.1	0.1	0	0	0	0	1	1
0.2	0.16	0.5	0.46	0.04	0.5	0.48	0.02	0.2	0.16	0.53	0.49	0.04	0.2	0.16	0.53	0.49	0.04	0.04	0	0	0	0	1	1

†Values shown in this section are the proportion of the simulated replicates for which the null model cannot be rejected, given the level of significance (0.95 or 0.99).

Supplementary Table 5: Speciation and extinction rates estimated from simulated data under the equal, compatible and incompatible rates models when a proportion (x) of extinct ranges are uniformly removed. Estimated parameters are averaged over 100 simulations under each parameter setting.

True parameter values					Equal rates			Compatible rates					Incompatible rates						Model comparison†					
																			Eq. v Co.		Eq. v In.		Co. v In.	
λ	μ	λ^*	μ^*	$d = d^*$	$\hat{\lambda} = \hat{\lambda}^*$	$\hat{\mu} = \hat{\mu}^*$	$\hat{d} = \hat{d}^*$	$\hat{\lambda}$	$\hat{\mu}$	$\hat{\lambda}^*$	$\hat{\mu}^*$	$\hat{d} = \hat{d}^*$	$\hat{\lambda}$	$\hat{\mu}$	$\hat{\lambda}^*$	$\hat{\mu}^*$	$\hat{d}$	$\hat{d}^*$	.95	.99	.95	.99	.95	.99
**Proportion of missing extinct ranges $x = 0.1$**																								
$\beta = 0, \lambda_a = 0$																								
0.2	0.02	0.2	0.02	0.18	0.2	0.02	0.18	0.2	0.01	0.2	0.02	0.18	0.2	0.03	0.2	0.02	0.18	0.18	1	1	1	1	1	1
0.2	0.1	0.2	0.1	0.1	0.2	0.09	0.1	0.19	0.09	0.2	0.09	0.1	0.2	0.1	0.2	0.09	0.1	0.11	0.98	1	0.99	1	0.96	0.99
0.2	0.16	0.2	0.16	0.04	0.2	0.16	0.04	0.19	0.15	0.2	0.16	0.05	0.2	0.15	0.2	0.16	0.04	0.05	0.94	1	1	1	0.99	1
$\beta = 0.5, \lambda_a = 0.04$																								
0.2	0.02	0.34	0.16	0.18	0.31	0.15	0.16	0.2	0.02	0.34	0.15	0.19	0.2	0.03	0.34	0.15	0.18	0.19	0	0.01	0	0.11	1	1
0.2	0.1	0.34	0.24	0.1	0.32	0.23	0.08	0.2	0.1	0.33	0.23	0.1	0.2	0.1	0.34	0.23	0.1	0.11	0	0.02	0	0.17	1	1
0.2	0.16	0.34	0.3	0.04	0.33	0.3	0.03	0.2	0.15	0.34	0.3	0.05	0.2	0.15	0.34	0.3	0.04	0.05	0	0	0	0.06	1	1
$\beta = 0.7, \lambda_a = 0.16$																								
0.2	0.02	0.5	0.32	0.18	0.45	0.32	0.13	0.2	0.02	0.5	0.31	0.19	0.2	0.03	0.5	0.31	0.18	0.19	0	0	0	0	1	1
0.2	0.1	0.5	0.4	0.1	0.46	0.4	0.07	0.2	0.1	0.5	0.39	0.1	0.2	0.1	0.5	0.39	0.1	0.11	0	0	0	0	1	1
0.2	0.16	0.5	0.46	0.04	0.49	0.46	0.03	0.2	0.15	0.5	0.46	0.04	0.2	0.15	0.5	0.46	0.04	0.05	0	0	0	0	1	1
**Proportion of missing extinct ranges $x = 0.5$**																								
$\beta = 0, \lambda_a = 0$																								
0.2	0.02	0.2	0.02	0.18	0.2	0.01	0.19	0.2	0.01	0.2	0.01	0.19	0.2	0.03	0.2	0.01	0.18	0.19	1	1	1	1	0.99	1
0.2	0.1	0.2	0.1	0.1	0.19	0.07	0.12	0.18	0.06	0.19	0.07	0.12	0.2	0.1	0.2	0.07	0.1	0.13	0.96	1	0.9	0.99	0.72	0.91
0.2	0.16	0.2	0.16	0.04	0.19	0.14	0.06	0.18	0.13	0.19	0.14	0.06	0.2	0.16	0.2	0.13	0.04	0.07	0.94	1	0.86	0.98	0.63	0.88
$\beta = 0.5, \lambda_a = 0.04$																								
0.2	0.02	0.34	0.16	0.18	0.29	0.11	0.18	0.2	0	0.32	0.11	0.2	0.2	0.03	0.34	0.1	0.18	0.24	0	0.01	0	0	0.62	0.94
0.2	0.1	0.34	0.24	0.1	0.3	0.2	0.1	0.18	0.06	0.32	0.2	0.13	0.2	0.1	0.34	0.18	0.1	0.15	0	0.03	0	0	0.51	0.85
0.2	0.16	0.34	0.3	0.04	0.32	0.28	0.04	0.19	0.14	0.33	0.28	0.05	0.2	0.16	0.34	0.27	0.04	0.07	0	0.01	0	0	0.7	0.98
$\beta = 0.7, \lambda_a = 0.16$																								
0.2	0.02	0.5	0.32	0.18	0.41	0.26	0.15	0.2	0	0.46	0.26	0.2	0.2	0.03	0.5	0.24	0.18	0.27	0	0	0	0	0.02	0.78
0.2	0.1	0.5	0.4	0.1	0.43	0.35	0.08	0.19	0.06	0.47	0.35	0.12	0.2	0.1	0.5	0.33	0.1	0.17	0	0	0	0	0.42	0.85
0.2	0.16	0.5	0.46	0.04	0.47	0.44	0.03	0.19	0.14	0.49	0.44	0.05	0.2	0.16	0.5	0.43	0.04	0.08	0	0	0	0	0.83	0.99

– table continued on the next page

Table continued from the previous page.

																			Model comparison†					
True parameter values					Equal rates			Compatible rates					Incompatible rates						Eq. v Co.		Eq. v In.		Co. v In.	
λ	μ	λ^*	μ^*	$d = d^*$	$\hat{\lambda} = \hat{\lambda}^*$	$\hat{\mu} = \hat{\mu}^*$	$\hat{d} = \hat{d}^*$	$\hat{\lambda}$	$\hat{\mu}$	$\hat{\lambda}^*$	$\hat{\mu}^*$	$\hat{d} = \hat{d}^*$	$\hat{\lambda}$	$\hat{\mu}$	$\hat{\lambda}^*$	$\hat{\mu}^*$	$\hat{d}$	$\hat{d}^*$	.95	.99	.95	.99	.95	.99
**Proportion of missing extinct ranges $x = 0.9$**																								
$\beta = 0, \lambda_a = 0$																								
0.2	0.02	0.2	0.02	0.18	0.2	0	0.19	0.2	0	0.2	0	0.19	0.2	0.03	0.2	0	0.18	0.2	1	1	1	1	0.96	1
0.2	0.1	0.2	0.1	0.1	0.17	0.02	0.15	0.17	0.01	0.18	0.02	0.16	0.2	0.1	0.2	0.02	0.1	0.18	1	1	0.08	0.27	0.05	0.15
0.2	0.16	0.2	0.16	0.04	0.16	0.08	0.09	0.16	0.07	0.16	0.08	0.09	0.2	0.16	0.2	0.06	0.04	0.14	0.97	1	0	0	0	0
$\beta = 0.5, \lambda_a = 0.04$																								
0.2	0.02	0.34	0.16	0.18	0.26	0.03	0.22	0.23	0	0.27	0.04	0.23	0.2	0.03	0.34	0.03	0.18	0.32	0	0.29	0	0	0	0
0.2	0.1	0.34	0.24	0.1	0.24	0.09	0.15	0.18	0.01	0.26	0.09	0.17	0.2	0.1	0.34	0.07	0.1	0.27	0.01	0.11	0	0	0	0
0.2	0.16	0.34	0.3	0.04	0.26	0.19	0.06	0.17	0.1	0.27	0.19	0.08	0.2	0.16	0.34	0.15	0.04	0.19	0	0.19	0	0	0	0
$\beta = 0.7, \lambda_a = 0.16$																								
0.2	0.02	0.5	0.32	0.18	0.33	0.11	0.22	0.23	0	0.36	0.13	0.23	0.2	0.03	0.5	0.08	0.18	0.43	0	0	0	0	0	0
0.2	0.1	0.5	0.4	0.1	0.33	0.21	0.13	0.18	0.01	0.37	0.21	0.16	0.2	0.1	0.5	0.14	0.1	0.36	0	0	0	0	0	0
0.2	0.16	0.5	0.46	0.04	0.38	0.33	0.04	0.18	0.11	0.41	0.34	0.07	0.2	0.16	0.5	0.27	0.04	0.23	0	0	0	0	0	0

†Values shown in this section are the proportion of the simulated replicates for which the null model cannot be rejected, given the level of significance (0.95 or 0.99).

Supplementary Table 6: Speciation and extinction rates estimated from simulated data under the equal, compatible and incompatible rates models when a proportion (x) of extant ranges are uniformly removed. Estimated parameters are averaged over 100 simulations under each parameter setting.

																			Model comparison†					
True parameter values					Equal rates			Compatible rates					Incompatible rates						Eq. v Co.		Eq. v In.		Co. v In.	
λ	μ	λ^*	μ^*	$d = d^*$	$\hat{\lambda} = \hat{\lambda}^*$	$\hat{\mu} = \hat{\mu}^*$	$\hat{d} = \hat{d}^*$	$\hat{\lambda}$	$\hat{\mu}$	$\hat{\lambda}^*$	$\hat{\mu}^*$	$\hat{d} = \hat{d}^*$	$\hat{\lambda}$	$\hat{\mu}$	$\hat{\lambda}^*$	$\hat{\mu}^*$	$\hat{d}$	$\hat{d}^*$	.95	.99	.95	.99	.95	.99
**Proportion of missing extant ranges $x = 0.1$**																								
$\beta = 0, \lambda_a = 0$																								
0.2	0.02	0.2	0.02	0.18	0.2	0.02	0.18	0.2	0.02	0.2	0.02	0.18	0.2	0.03	0.2	0.02	0.18	0.18	1	1	1	1	1	1
0.2	0.1	0.2	0.1	0.1	0.2	0.1	0.1	0.19	0.09	0.2	0.1	0.1	0.2	0.09	0.2	0.1	0.1	0.1	0.95	1	0.99	1	0.98	1
0.2	0.16	0.2	0.16	0.04	0.2	0.16	0.04	0.19	0.15	0.2	0.16	0.04	0.2	0.16	0.2	0.16	0.04	0.04	0.98	1	1	1	0.98	1
$\beta = 0.5, \lambda_a = 0.04$																								
0.2	0.02	0.34	0.16	0.18	0.31	0.17	0.15	0.2	0.03	0.34	0.17	0.18	0.2	0.03	0.34	0.17	0.18	0.17	0	0.03	0.03	0.18	1	1
0.2	0.1	0.34	0.24	0.1	0.32	0.25	0.08	0.2	0.1	0.34	0.24	0.1	0.2	0.09	0.34	0.25	0.1	0.09	0	0.02	0.02	0.13	1	1
0.2	0.16	0.34	0.3	0.04	0.33	0.3	0.03	0.2	0.16	0.34	0.3	0.04	0.2	0.16	0.34	0.3	0.04	0.04	0	0.03	0.04	0.08	1	1
$\beta = 0.7, \lambda_a = 0.16$																								
0.2	0.02	0.5	0.32	0.18	0.46	0.34	0.12	0.2	0.03	0.51	0.33	0.18	0.2	0.03	0.5	0.33	0.18	0.17	0	0	0	0	1	1
0.2	0.1	0.5	0.4	0.1	0.47	0.41	0.06	0.2	0.1	0.5	0.4	0.1	0.2	0.09	0.5	0.41	0.1	0.09	0	0	0	0	1	1
0.2	0.16	0.5	0.46	0.04	0.49	0.46	0.02	0.2	0.16	0.5	0.46	0.04	0.2	0.16	0.5	0.46	0.04	0.04	0	0	0	0	1	1
**Proportion of missing extant ranges $x = 0.5$**																								
$\beta = 0, \lambda_a = 0$																								
0.2	0.02	0.2	0.02	0.18	0.21	0.03	0.17	0.2	0.03	0.21	0.03	0.17	0.2	0.03	0.2	0.04	0.18	0.17	1	1	1	1	1	1
0.2	0.1	0.2	0.1	0.1	0.21	0.13	0.08	0.2	0.12	0.21	0.13	0.08	0.2	0.1	0.2	0.13	0.1	0.07	0.99	1	0.93	0.99	0.87	0.98
0.2	0.16	0.2	0.16	0.04	0.2	0.17	0.03	0.2	0.17	0.21	0.17	0.03	0.2	0.16	0.2	0.18	0.04	0.02	0.98	1	0.95	1	0.91	0.99
$\beta = 0.5, \lambda_a = 0.04$																								
0.2	0.02	0.34	0.16	0.18	0.33	0.21	0.12	0.22	0.06	0.36	0.21	0.15	0.2	0.03	0.34	0.22	0.18	0.12	0	0.06	0.06	0.18	0.81	1
0.2	0.1	0.34	0.24	0.1	0.33	0.27	0.06	0.21	0.13	0.35	0.27	0.08	0.2	0.1	0.34	0.28	0.1	0.06	0	0.08	0.07	0.17	0.85	0.98
0.2	0.16	0.34	0.3	0.04	0.34	0.31	0.02	0.21	0.17	0.35	0.31	0.03	0.2	0.16	0.34	0.32	0.04	0.02	0.01	0.04	0.04	0.11	0.99	1
$\beta = 0.7, \lambda_a = 0.16$																								
0.2	0.02	0.5	0.32	0.18	0.47	0.38	0.09	0.22	0.06	0.53	0.37	0.15	0.2	0.03	0.5	0.39	0.18	0.11	0	0	0	0	0.69	1
0.2	0.1	0.5	0.4	0.1	0.48	0.43	0.05	0.21	0.13	0.51	0.43	0.08	0.2	0.1	0.5	0.44	0.1	0.06	0	0	0	0	0.9	0.99
0.2	0.16	0.5	0.46	0.04	0.49	0.48	0.02	0.2	0.17	0.51	0.47	0.03	0.2	0.16	0.5	0.48	0.04	0.02	0	0	0	0	0.99	1

– table continued on the next page

Table continued from the previous page.

True parameter values					Equal rates			Compatible rates					Incompatible rates						Model comparison†					
																			Eq. v Co.		Eq. v In.		Co. v In.	
λ	μ	λ^*	μ^*	$d = d^*$	$\hat{\lambda} = \hat{\lambda}^*$	$\hat{\mu} = \hat{\mu}^*$	$\hat{d} = \hat{d}^*$	$\hat{\lambda}$	$\hat{\mu}$	$\hat{\lambda}^*$	$\hat{\mu}^*$	$\hat{d} = \hat{d}^*$	$\hat{\lambda}$	$\hat{\mu}$	$\hat{\lambda}^*$	$\hat{\mu}^*$	$\hat{d}$	$\hat{d}^*$	.95	.99	.95	.99	.95	.99
**Proportion of missing extant ranges $x = 0.9$**																								
$\beta = 0, \lambda_a = 0$																								
0.2	0.02	0.2	0.02	0.18	0.23	0.09	0.15	0.21	0.05	0.25	0.09	0.16	0.2	0.03	0.2	0.11	0.18	0.1	0.77	0.92	0.67	0.94	0.55	0.89
0.2	0.1	0.2	0.1	0.1	0.22	0.16	0.06	0.22	0.15	0.23	0.16	0.06	0.2	0.11	0.2	0.18	0.1	0.02	0.96	0.99	0.01	0.32	0	0.04
0.2	0.16	0.2	0.16	0.04	0.21	0.19	0.02	0.2	0.18	0.21	0.19	0.02	0.2	0.16	0.2	0.2	0.04	0.01	0.93	1	0.42	0.77	0.02	0.54
$\beta = 0.5, \lambda_a = 0.04$																								
0.2	0.02	0.34	0.16	0.18	0.35	0.27	0.09	0.23	0.1	0.4	0.27	0.13	0.2	0.03	0.34	0.31	0.18	0.03	0.02	0.08	0	0	0	0
0.2	0.1	0.34	0.24	0.1	0.35	0.3	0.04	0.23	0.16	0.37	0.3	0.06	0.2	0.11	0.34	0.33	0.1	0.01	0.01	0.09	0	0.06	0	0.16
0.2	0.16	0.34	0.3	0.04	0.34	0.33	0.02	0.21	0.18	0.35	0.33	0.03	0.2	0.16	0.34	0.34	0.04	0	0	0.02	0	0.04	0.29	0.77
$\beta = 0.7, \lambda_a = 0.16$																								
0.2	0.02	0.5	0.32	0.18	0.5	0.43	0.07	0.23	0.1	0.56	0.43	0.13	0.2	0.03	0.5	0.48	0.18	0.03	0	0	0	0	0	0
0.2	0.1	0.5	0.4	0.1	0.49	0.46	0.03	0.22	0.15	0.53	0.46	0.07	0.2	0.11	0.5	0.49	0.1	0.01	0	0	0	0	0	0.26
0.2	0.16	0.5	0.46	0.04	0.5	0.49	0.01	0.21	0.18	0.51	0.48	0.03	0.2	0.16	0.5	0.5	0.04	0	0	0	0	0	0.52	0.9

†Values shown in this section are the proportion of the simulated replicates for which the null model cannot be rejected, given the level of significance (0.95 or 0.99).

Supplementary Table 7: Speciation and extinction rates estimated from simulated data under the equal, compatible and incompatible rates models when diversification rates vary, with a peak in diversification during the interval 10-20 time units. Estimated parameters are averaged over 100 simulations under each parameter setting.

True parameter values*				Equal rates			Compatible rates					Incompatible rates						Model comparison†					
																		Eq. v Co.		Eq. v In.		Co. v In.	
λ	μ	λ^*	μ^*	$\hat{\lambda} = \hat{\lambda}^*$	$\hat{\mu} = \hat{\mu}^*$	$\hat{d} = \hat{d}^*$	$\hat{\lambda}$	$\hat{\mu}$	$\hat{\lambda}^*$	$\hat{\mu}^*$	$\hat{d} = \hat{d}^*$	$\hat{\lambda}$	$\hat{\mu}$	$\hat{\lambda}^*$	$\hat{\mu}^*$	$\hat{d}$	$\hat{d}^*$	.95	.99	.95	.99	.95	.99
$\beta = 0, \lambda_a = 0$																							
0.2 (0.3)	0.02 (0)	0.2 (0.3)	0.02 (0)	0.21	0.02	0.19	0.2	0.01	0.21	0.02	0.19	0.2	0.01	0.21	0.02	0.2	0.19	1	1	1	1	1	1
0.2 (0.3)	0.1 (0)	0.2 (0.3)	0.1 (0)	0.22	0.08	0.13	0.19	0.05	0.22	0.08	0.14	0.18	0.02	0.22	0.09	0.16	0.13	0.69	0.97	0.82	0.98	0.9	1
0.2 (0.3)	0.16 (0)	0.2 (0.3)	0.16 (0)	0.22	0.13	0.1	0.18	0.07	0.23	0.13	0.1	0.16	0.03	0.22	0.13	0.13	0.09	0.33	0.74	0.38	0.63	0.45	0.88
$\beta = 0.5, \lambda_a = 0.04$																							
0.2 (0.3)	0.02 (0)	0.34 (0.49)	0.16 (0.19)	0.33	0.16	0.16	0.2	0.01	0.36	0.16	0.2	0.2	0.01	0.35	0.16	0.2	0.19	0	0	0	0.03	1	1
0.2 (0.3)	0.1 (0)	0.34 (0.49)	0.24 (0.19)	0.34	0.23	0.11	0.19	0.04	0.37	0.23	0.15	0.18	0.02	0.36	0.23	0.16	0.13	0	0.01	0.01	0.03	1	1
0.2 (0.3)	0.16 (0)	0.34 (0.49)	0.3 (0.19)	0.36	0.28	0.08	0.17	0.06	0.38	0.27	0.11	0.16	0.03	0.37	0.28	0.13	0.09	0	0	0	0	0.62	0.96
$\beta = 0.7, \lambda_a = 0.16$																							
0.2 (0.3)	0.02 (0)	0.5 (0.67)	0.32 (0.37)	0.46	0.33	0.14	0.2	0.01	0.52	0.32	0.2	0.2	0.01	0.51	0.32	0.2	0.19	0	0	0	0	1	1
0.2 (0.3)	0.1 (0)	0.5 (0.67)	0.4 (0.37)	0.49	0.4	0.09	0.18	0.04	0.54	0.39	0.15	0.18	0.02	0.53	0.4	0.16	0.13	0	0	0	0	1	1
0.2 (0.3)	0.16 (0)	0.5 (0.67)	0.46 (0.37)	0.51	0.44	0.07	0.17	0.06	0.55	0.43	0.12	0.16	0.03	0.53	0.44	0.13	0.09	0	0	0	0	0.82	1

†Values shown in this section are the proportion of the simulated replicates for which the null model cannot be rejected, given the level of significance (0.95 or 0.99). *Numbers in brackets show the rates during the interval 10–20.

Supplementary Table 8: Speciation and extinction rates estimated from simulated data under the equal and compatible and incompatible rates models when diversification rates vary. Results are shown for (1) a scenario in which an interval of high diversification is follow by an interval of equal speciation and extinction $\lambda = \mu$ and (2) a scenario in which diversification decreases over time and $\lambda < \mu$ during the final interval.

True parameter values*								Equal	Compatible	Incompatible		Model comparison†					
												Eq. v Co.		Eq. v In.		Co. v In.	
λ	μ	d	β	λ_a	λ^*	μ^*	d^*	$\hat{d} = \hat{d}^*$	$\hat{d} = \hat{d}^*$	$\hat{d}$	$\hat{d}^*$	.95	.99	.95	.99	.95	.99
0.2,0.3,0.2	0.02,0,0.2	0.18,0.3,0	0	0	0.2,0.3,0.2	0.02,0,0.2	0.18,0.3,0	0.06	0.07	0.09	0.05	0.19	0.59	0.26	0.48	0.36	0.82
0.2,0.3,0.2	0.02,0,0.2	0.18,0.3,0	0.5	0.04	0.34,0.49,0.34	0.16,0.19,0.34	0.18,0.3,0	0.05	0.07	0.09	0.05	0	0	0	0	0.59	0.94
0.2,0.3,0.2	0.02,0,0.2	0.18,0.3,0	0.7	0.16	0.50,0.67,0.50	0.32,0.37,0.50	0.18,0.3,0	0.04	0.08	0.09	0.05	0	0	0	0	0.75	0.98
0.3,0.2,0.2	0.02,0.01,0.30	0.28, 0.19,-0.10	0	0	0.3,0.2,0.2	0.02,0.01,0.30	0.28, 0.19,-0.10	0.02	0.03	0.06	0.02	0.03	0.27	0.02	0.03	0.01	0.14
0.3,0.2,0.2	0.02,0.01,0.30	0.28, 0.19,-0.10	0.5	0.04	0.49,0.34,0.34	0.21,0.15,0.44	0.28, 0.19,-0.10	0.02	0.03	0.06	0.02	0	0	0	0	0.02	0.23
0.3,0.2,0.2	0.02,0.01,0.30	0.28, 0.19,-0.10	0.7	0.16	0.67,0.50,0.50	0.39,0.31,0.60	0.28, 0.19,-0.10	0.02	0.04	0.06	0.02	0	0	0	0	0.06	0.39

†Values shown in this section are the proportion of the simulated replicates for which the null model cannot be rejected, given the level of significance (0.95 or 0.99). *Birth-death parameters in descending order from oldest to youngest, prior to 25 time units, and during the intervals 15–25 and 0–15.

Supplementary Table 9: Speciation and extinction rates estimated from simulated data under the equal, compatible and incompatible rates models when a proportion (κ) of speciation events are cryptic. Estimated parameters are averaged over 100 simulations under each parameter setting.

																			Model comparison†					
True parameter values					Equal rates			Compatible rates					Incompatible rates						Eq. v Co.		Eq. v In.		Co. v In.	
λ	μ	λ^*	μ^*	$d = d^*$	$\hat{\lambda} = \hat{\lambda}^*$	$\hat{\mu} = \hat{\mu}^*$	$\hat{d} = \hat{d}^*$	$\hat{\lambda}$	$\hat{\mu}$	$\hat{\lambda}^*$	$\hat{\mu}^*$	$\hat{d} = \hat{d}^*$	$\hat{\lambda}$	$\hat{\mu}$	$\hat{\lambda}^*$	$\hat{\mu}^*$	$\hat{d}$	$\hat{d}^*$	.95	.99	.95	.99	.95	.99
$\kappa = \mathbf{0.1}$																								
$\beta = 0, \lambda_a = 0$																								
0.2	0.02	0.2	0.02	0.18	0.2	0.02	0.18	0.19	0.01	0.2	0.02	0.18	0.2	0.03	0.2	0.02	0.18	0.18	1	1	1	1	1	1
0.2	0.1	0.2	0.1	0.1	0.19	0.09	0.1	0.19	0.08	0.19	0.09	0.1	0.2	0.1	0.19	0.09	0.1	0.1	0.99	1	0.96	1	0.95	0.98
0.2	0.16	0.2	0.16	0.04	0.18	0.14	0.04	0.18	0.14	0.19	0.14	0.04	0.2	0.15	0.18	0.14	0.04	0.04	1	1	0.97	0.98	0.95	0.98
$\beta = 0.5, \lambda_a = 0.04$																								
0.2	0.02	0.34	0.16	0.18	0.27	0.11	0.16	0.2	0.02	0.29	0.11	0.18	0.2	0.03	0.29	0.11	0.18	0.18	0.05	0.4	0.48	0.81	1	1
0.2	0.1	0.34	0.24	0.1	0.27	0.19	0.09	0.2	0.1	0.29	0.19	0.1	0.2	0.1	0.29	0.19	0.1	0.1	0.08	0.38	0.42	0.71	1	1
0.2	0.16	0.34	0.3	0.04	0.28	0.25	0.04	0.2	0.16	0.29	0.25	0.04	0.2	0.15	0.29	0.25	0.04	0.04	0.04	0.22	0.28	0.58	1	1
$\beta = 0.7, \lambda_a = 0.16$																								
0.2	0.02	0.5	0.32	0.18	0.37	0.24	0.14	0.2	0.02	0.41	0.23	0.18	0.2	0.03	0.41	0.23	0.18	0.18	0	0	0	0	1	1
0.2	0.1	0.5	0.4	0.1	0.38	0.31	0.07	0.2	0.1	0.41	0.31	0.1	0.2	0.1	0.41	0.31	0.1	0.1	0	0	0	0	1	1
0.2	0.16	0.5	0.46	0.04	0.4	0.37	0.03	0.2	0.16	0.41	0.37	0.04	0.2	0.15	0.41	0.37	0.04	0.04	0	0	0	0.02	1	1
$\kappa = \mathbf{0.5}$																								
$\beta = 0, \lambda_a = 0$																								
0.2	0.02	0.2	0.02	0.18	0.19	0.01	0.18	0.19	0.01	0.2	0.01	0.18	0.2	0.03	0.19	0.01	0.18	0.18	1	1	1	1	0.97	1
0.2	0.1	0.2	0.1	0.1	0.17	0.07	0.11	0.17	0.07	0.17	0.07	0.11	0.2	0.1	0.17	0.07	0.1	0.1	1	1	0.9	0.96	0.81	0.92
0.2	0.16	0.2	0.16	0.04	0.16	0.11	0.05	0.16	0.11	0.16	0.11	0.05	0.2	0.15	0.15	0.11	0.04	0.04	1	1	0.81	0.92	0.53	0.84
$\beta = 0.5, \lambda_a = 0.04$																								
0.2	0.02	0.34	0.16	0.18	0.22	0.05	0.17	0.2	0.02	0.23	0.05	0.18	0.2	0.03	0.23	0.05	0.18	0.18	0.83	1	1	1	1	1
0.2	0.1	0.34	0.24	0.1	0.21	0.12	0.1	0.2	0.1	0.22	0.12	0.1	0.2	0.1	0.22	0.12	0.1	0.1	0.92	1	0.99	1	0.99	1
0.2	0.16	0.34	0.3	0.04	0.21	0.17	0.04	0.2	0.15	0.21	0.17	0.04	0.2	0.15	0.21	0.17	0.04	0.04	0.83	0.99	0.99	1	0.98	1
$\beta = 0.7, \lambda_a = 0.16$																								
0.2	0.02	0.5	0.32	0.18	0.28	0.12	0.16	0.2	0.02	0.3	0.12	0.18	0.2	0.03	0.3	0.12	0.18	0.18	0.04	0.33	0.4	0.72	1	1
0.2	0.1	0.5	0.4	0.1	0.27	0.19	0.09	0.2	0.1	0.29	0.19	0.1	0.2	0.1	0.29	0.19	0.1	0.1	0.09	0.43	0.46	0.67	1	1
0.2	0.16	0.5	0.46	0.04	0.28	0.24	0.04	0.2	0.16	0.29	0.24	0.04	0.2	0.15	0.28	0.24	0.04	0.04	0.05	0.28	0.29	0.63	1	1

– table continued on the next page

Table continued from the previous page.

																			Model comparison†					
True parameter values					Equal rates			Compatible rates					Incompatible rates						Eq. v Co.		Eq. v In.		Co. v In.	
λ	μ	λ^*	μ^*	$d = d^*$	$\hat{\lambda} = \hat{\lambda}^*$	$\hat{\mu} = \hat{\mu}^*$	$\hat{d} = \hat{d}^*$	$\hat{\lambda}$	$\hat{\mu}$	$\hat{\lambda}^*$	$\hat{\mu}^*$	$\hat{d} = \hat{d}^*$	$\hat{\lambda}$	$\hat{\mu}$	$\hat{\lambda}^*$	$\hat{\mu}^*$	$\hat{d}$	$\hat{d}^*$	.95	.99	.95	.99	.95	.99
$\kappa = \mathbf{0.9}$																								
$\beta = 0, \lambda_a = 0$																								
0.2	0.02	0.2	0.02	0.18	0.19	0.01	0.18	0.19	0.01	0.19	0.01	0.18	0.2	0.03	0.16	0.01	0.18	0.15	1	1	0.94	0.98	0.86	0.96
0.2	0.1	0.2	0.1	0.1	0.16	0.05	0.12	0.16	0.05	0.16	0.05	0.12	0.2	0.1	0.13	0.04	0.1	0.09	1	1	0.59	0.8	0.45	0.63
0.2	0.16	0.2	0.16	0.04	0.13	0.07	0.06	0.13	0.07	0.13	0.07	0.06	0.2	0.15	0.11	0.07	0.04	0.04	1	1	0.08	0.22	0.06	0.09
$\beta = 0.5, \lambda_a = 0.04$																								
0.2	0.02	0.34	0.16	0.18	0.19	0.01	0.18	0.19	0.01	0.19	0.01	0.18	0.2	0.03	0.18	0.01	0.18	0.16	1	1	0.98	0.98	0.93	0.98
0.2	0.1	0.34	0.24	0.1	0.16	0.05	0.11	0.16	0.05	0.16	0.05	0.11	0.2	0.1	0.15	0.05	0.1	0.09	1	1	0.74	0.92	0.57	0.79
0.2	0.16	0.34	0.3	0.04	0.13	0.08	0.06	0.13	0.08	0.13	0.08	0.06	0.2	0.15	0.12	0.08	0.04	0.04	1	1	0.18	0.38	0.08	0.2
$\beta = 0.7, \lambda_a = 0.16$																								
0.2	0.02	0.5	0.32	0.18	0.2	0.02	0.18	0.19	0.01	0.2	0.02	0.18	0.2	0.03	0.19	0.02	0.18	0.17	0.99	1	1	1	0.96	1
0.2	0.1	0.5	0.4	0.1	0.17	0.06	0.11	0.17	0.06	0.17	0.06	0.11	0.2	0.1	0.16	0.06	0.1	0.1	1	1	0.8	0.93	0.63	0.84
0.2	0.16	0.5	0.46	0.04	0.14	0.09	0.05	0.14	0.09	0.14	0.09	0.05	0.2	0.15	0.13	0.09	0.04	0.04	1	1	0.36	0.59	0.24	0.39

†Values shown in this section are the proportion of the simulated replicates for which the null model cannot be rejected, given the level of significance (0.95 or 0.99).

Supplementary Table 10: Speciation and extinction rates estimated from empirical datasets of extant phylogenies and stratigraphic ranges under the equal, compatible and incompatible rates models.

																		Model comparison†					
	Equal rates			Compatible rates					Incompatible rates						Likelihood			Eq. v Co.		Eq. v In.		Co. v In.	
Clade	$\hat{\lambda} = \hat{\lambda}^*$	$\hat{\mu} = \hat{\mu}^*$	$\hat{d} = \hat{d}^*$	$\hat{\lambda}$	$\hat{\mu}$	$\hat{\lambda}^*$	$\hat{\mu}^*$	$\hat{d} = \hat{d}^*$	$\hat{\lambda}$	$\hat{\mu}$	$\hat{\lambda}^*$	$\hat{\mu}^*$	$\hat{d}$	$\hat{d}^*$	$L_{Eq.}$	$L_{Co.}$	$L_{In.}$	.95	.99	.95	.99	.95	.99
Bovidae	0.39	0.34	0.06	0.12	0	0.45	0.33	0.12	0.12	0	0.42	0.35	0.12	0.07	-2410	$-2368^{\star}$	$-2366^{\diamond}$	0	0	0	0	1	1
Canidae	0.26	0.15	0.11	0.14	0.01	0.28	0.15	0.13	0.15	0	0.27	0.15	0.15	0.12	$-780^{\star}$	$-777^{\diamond}$	-777	0	0	0	1	1	1
Cervidae	0.51	0.41	0.1	0.29	0.15	0.54	0.4	0.14	0.22	0	0.5	0.44	0.22	0.06	-820	$-818^{\star}$	$-815^{\diamond}$	0	0	0	0	0	1
Cetacea	0.34	0.3	0.04	0.12	0.06	0.36	0.29	0.07	0.11	0	0.34	0.31	0.11	0.04	-3349	-3333	$-3330^{\star\diamond}$	0	0	0	0	0	1
Feliformia	0.28	0.2	0.08	0.12	0.01	0.31	0.2	0.11	0.12	0.01	0.31	0.2	0.11	0.12	-2237	$-2220^{\star\diamond}$	-2220	0	0	0	0	1	1
Ferns	0.02	0.01	0.01	0.01	0.01	0.02	0.01	0.01	0.01	0	0.02	0.01	0.01	0	-5235	-5229	$-5218^{\star\diamond}$	0	0	0	0	0	0
Scleractinia	0.13	0.12	0.01	0.13	0.12	0.13	0.12	0.01	0.52	0.51	0.13	0.12	0.01	0.01	-29175	-29175	$-29139^{\star\diamond}$	1	1	0	0	0	0
Sphenisciformes	0.27	0.23	0.04	0.27	0.23	0.27	0.23	0.04	0.3	0.2	0.25	0.23	0.1	0.02	$-405^{\star\diamond}$	-405	-404	1	1	1	1	1	1
Ursidae	0.34	0.34	0	0.11	0.07	0.36	0.33	0.04	0.1	0.04	0.36	0.33	0.06	0.03	$-320^{\star}$	$-318^{\diamond}$	-318	0	1	1	1	1	1

†Values shown in this section indicate whether the null model was accepted (1) or rejected (0), given the level of significance (0.95 or 0.99). Likelihood values are shown for the equal ($L_{Eq.}$), compatible ($L_{Co.}$) and incompatible ($L_{In.}$) rates models. The corresponding likelihood ratio values are shown in Table 12. * and $^{\diamond}$ indicate the best model at the 1% and 5% levels, respectively.

Supplementary Table 11: Parameter estimates under the BDC model applied to seven empirical clades, assuming constant diversification rates over time. The BDC model assumed equal rates for fossil and phylogenetic data for Canidae, Sphenisciformes, and Ursidae, and compatible rates for the other clades (see also Fig. 3). The analyses were carried out using the Bayesian implementation of the method and the parameter values reported here represent the mean of the posterior samples with 95% credible intervals given in parentheses. The range of possible λ_a and β values are also shown, which can be obtained based on the properties of the BDC model (see main text). Under the equal rates model all speciation occurs via budding. Under the compatible rates model, anagenesis exceeds budding when $\lambda^* - 2\lambda > 0$, whereas budding exceeds anagenesis $\lambda^* - 2\lambda < 0$. The contribution of both processes will be equal when $\lambda^* - 2\lambda \approx 0$ (Fig. 4).

Clade	λ^*	μ^*	λ	μ	$d = d^*$	λ_a	β
Canidae	0.26 (0.23–0.29)	0.15 (0.12–0.18)	0.26 (0.23–0.29)	0.15 (0.12–0.18)	0.11 (0.07–0.15)	0	0
Sphenisciformes	0.27 (0.21–0.33)	0.23 (0.18–0.29)	0.27 (0.21–0.33)	0.23 (0.18–0.29)	0.04 (-0.03–0.10)	0	0
Ursidae	0.34 (0.27–0.41)	0.34 (0.27–0.41)	0.34 (0.27–0.41)	0.34 (0.27–0.41)	0 (-0.09–0.09)	0	0
Bovidae	0.44 (0.42–0.47)	0.33 (0.31–0.36)	0.12 (0.10–0.15)	0.02 (0–0.04)	0.11 (0.09–0.13)	(0.19–0.32)	(0–1)
Cervidae	0.54 (0.47–0.60)	0.40 (0.35–0.46)	0.31 (0.18–0.45)	0.18 (0.02–0.34)	0.13 (0.07–0.20)	(0.00–0.23)	(0–0.72)
Cetacea	0.36 (0.33-0.38)	0.29 (0.27–0.32)	0.13 (0.08–0.17)	0.06 (0.01–0.12)	0.06 (0.04–0.09)	(0.11–0.23)	(0–1)
Feliformia	0.31 (0.29–0.33)	0.20 (0.18–0.22)	0.13 (0.10–0.17)	0.03 (0–0.06)	0.11 (0.08–0.13)	(0.04–0.17)	(0–1)

Supplementary Table 12: Results of model testing between three birth-death models in empirical clades. The equal rates model (Eq.) assumes identical rates between phylogenetic and stratigraphic data; the compatible rates model (Co.) indicates that rates are significantly different, but compatible with different modes of speciation; the incompatible rates model (In.) assumes independent rate parameters. Results of model testing are given for both Bayesian and maximum likelihood implementations under thresholds of 0.05 and 0.01. The column *LR* shows the log likelihood ratio between the compatible and independent rates models. The fern dataset, re-analysed under the skyline model (with 6 rate shifts; see main text), provided support for the BDC model (compatible rates) in all 7 time bins at a 0.01 significance threshold (results reported in parentheses). In contrast, the Scleractinia dataset supported incompatible rates even under a skyline model.

	Likelihood ratio test			Bayesian analysis		Rate variation
Clade	$P < 0.05$	$P < 0.01$	LR	$P < 0.05$	$P < 0.01$	(fossils only)
Bovidae	In.	Co.	4.163	In.	Co.	6.703
Canidae	Co.	Eq.	0.961	Co.	Eq.	3.894
Cervidae	In.	Co.	5.694	In.	Co.	8.655
Cetacea	In.	In.	6.942	In.	Co.	5.6
Feliformia	Co.	Co.	0.052	Co.	Co.	2.036
Ferns*	In.	In.	20.931	In. (Co.)	In. (Co.)	17.738
Scleractinia*	In.	In.	72.68	In. (In.)	In. (In.)	43.334
Sphenisciformes	Eq.	Eq.	1.184	Eq.	Eq.	15.116
Ursidae	Co.	Eq.	0.091	Eq.	Eq.	4.8

Supplementary Table 13: Amount of rate variation in fossil data sets. Best model and probabilities of constant rates were obtained from sampling frequencies of RJMCMC, rate variation was computed as the ratio between the maximum and minimum rates marginalised in 1 Myr time bins.

Dataset	best model (n. λ^* rates)	Pr constant (λ^*)	λ^* variation (x-fold)	best model (n. μ^* rates)	Pr constant (μ^*)	μ^* variation (x-fold)
Bovidae	3	0.25	1.51	5	< 0.01	6.70
Canidae	1	0.38	1.65	2	< 0.01	3.89
Cervidae	2	< 0.01	2.56	3	< 0.01	8.66
Cetacea	3	< 0.01	3.28	5	< 0.01	5.60
Feliformia	2	< 0.01	2.04	2	0.17	1.57
Ferns	4	< 0.01	5.58	4	< 0.01	17.74
Scleractinia	5	< 0.01	39.17	6	< 0.01	43.33
Sphenisciformes	2	0.01	8.24	3	< 0.01	15.12
Ursidae	2	0.01	4.80	1	0.49	1.47

Supplementary Table 14: Model testing under maximum likelihood using simulated datasets based on the speciation and extinction rates estimated from the empirical phylogenies (λ and μ) and reflecting the number of lineages observed in the fossil record and phylogenetic trees. The results are based on 100 simulations for each clade. The equal rates model (Eq.) assumes identical rates between phylogenetic and stratigraphic data; the compatible rates model (Co.) indicates that rates are significantly different, but compatible with different modes of speciation; the incompatible rates model (In.) assumes independent rate parameters. Results are shown for the 95 and 99% confidence levels.

						Eq. v Co.		Eq. v In.		Co. v In.	
Clade	Tip #	Root age	Range #	Max range age	ρ	.95	.99	.95	.99	.95	.99
Bovidae	137	27.2	544	22.4	0.958	0	0	0	0	1	1
Canidae	35	16.3	173	39.8	1	0	0	0	0	1	1
Cervidae	46	19	209	22.6	0.5	0	0.2	0.2	0.5	1	1
Cetacea	87	35.9	761	55.3	0.978	0	0	0	0	1	1
Feliformia	121	53.2	483	37.4	1	0	0	0	0	1	1
Ferns	355	476	348	409.9	0.955	0	0	0	0	1	1
Scleractinia	120	247.3	4771	263.9	0.154	0	0.2	0.2	0.4	1	1
Sphenisciformes	19	12.7	77	62.3	1	0.4	0.8	0.9	1	1	1
Ursidae	8	17.2	75	23	1	0.1	0.2	0.3	0.5	1	1
